# *N*-Glycosylation of Lipocalin 2 Is Not Required for Secretion or Exosome Targeting

**DOI:** 10.3389/fphar.2018.00426

**Published:** 2018-04-25

**Authors:** Erawan Borkham-Kamphorst, Eddy Van de Leur, Steffen K. Meurer, Eva M. Buhl, Ralf Weiskirchen

**Affiliations:** ^1^Institute of Molecular Pathobiochemistry, Experimental Gene Therapy and Clinical Chemistry, RWTH University Hospital Aachen, Aachen, Germany; ^2^Institute of Pathology, Electron Microscopy Facility, RWTH University Hospital Aachen, Aachen, Germany

**Keywords:** *N*-glycan, lipocalins, neutrophils, CD81, Alix, inflammation, tunicamycin, thapsigargin

## Abstract

Lipocalin 2 (LCN2) is a highly conserved secreted adipokine acting as a serum transport protein for small hydrophobic molecules such as fatty acids and steroids. In addition, LCN2 limits bacterial growth by sequestering iron-containing siderophores and further protects against intestinal inflammation and tumorigenesis associated with alterations in the microbiota. Human LCN2 contains one *N*-glycosylation site conserved in other species. It was postulated that this post-translational modification could facilitate protein folding, protects from proteolysis, is required for proper trafficking from the Golgi apparatus to the cell surface, and might be relevant for effective secretion. We here show that the homologous nucleoside antibiotic tunicamycin blocks *N*-linked glycosylation but not secretion of LCN2 in primary murine hepatocytes, derivatives thereof, human lung carcinoma cell line A549, and human prostate cancer cell line PC-3. Moreover, both the glycosylated and the non-glycosylated LCN2 variants are equally targeted to exosomes, demonstrating that this post-translational modification is not necessary for proper trafficking of LCN2 into these membranous extracellular vesicles. Furthermore, a hydrophobic cluster analysis revealed that the *N*-glycosylation site is embedded in a highly hydrophobic evolutionarily conserved surrounding. In sum, our data indicate that the *N*-glycosylation of LCN2 is not required for proper secretion and exosome cargo recruitment in different cell types, but might be relevant to increase overall solubility.

## Introduction

In humans, ~15% of all genes are predicted to have at least one secreted protein product^1^. In sum, these proteins form the human protein secretome having important cellular and systemic functions in immune defense, intercellular communication, cell adhesion, cell differentiation, morphogenesis, and angiogenesis (Hathout, 2007). Moreover, some of these proteins are essential molecular mediators in metastasis, tumor angiogenesis, development of an inflammatory tumor microenvironment, and initiation or execution of cellular apoptosis, necrosis, and autophagy (Nikoletopoulou et al., 2013; Su et al., 2015). Therefore, the knowledge on mechanisms contributing to the secretion of proteins is important to understand their physiological roles and for their potential application in therapeutics. Proteins entering the secretory pathway are most often carrying a signal peptide with minimal requirements containing a hydrophobic core of 5–15 amino acids in length (von Heijne, 1985). In addition, eukaryotic secreted proteins contain several post-translational modifications. In particular, molecules involved in the innate and adaptive immune response are modulated by *N*-glycosylation (Rudd et al., 2001). This posttranslational modification contributes to the stability of the protein, enhances folding efficiency, prevents aggregation, facilitates formation of proper disulfide bonds, helps to orient binding forces, and provides protease protection. In some cases, these carbohydrates are necessary to restrict lateral protein-protein interaction between T cells and antigen-presenting cells (Rudd et al., 2001). As a consequence, aberrant or altered *N*-glycosylation is often associated with diseases and disorders. Prototypically this is illustrated in the biological consequences resulting from alterations within a single *N*-glycosylation site of the prostate-specific antigen (PSA) leading to prostate cancer development or faster disease progression (Drake et al., 2015).

LCN2, also known as neutrophil gelatinase-associated protein (NGAL), 24p3, siderocalin or uterocalin, was initially purified to apparent homogeneity from exocytosed material from phorbol myristate acetate-stimulated neutrophils (Kjeldsen et al., 1993). In this pioneering study, a single functional *N*-glycosylation site was identified sharing the consensus sequence for *N*-glycosylation (Asn-Xaa-Ser/Thr) and being susceptible to *N*-glycanase (Kjeldsen et al., 1993). Similarly, *N*-linked carbohydrates were identified in the murine homolog 24p3 (Chu et al., 1996). Concordantly, these former biochemical studies show that LCN2 is a glycosylated protein. However, data on functional aspects of LCN2 glycosylation is missing.

More recent work has demonstrated that conserved glycosylation signatures composed of complex *N*-linked glycans are enriched in exosomes and microvesicles (Batista et al., 2011). These nanosized membranous extracellular vesicles are secreted from most mammalian cell types, in all body fluids, and in all species tested so far (Lawson et al., 2017). They are typically composed of a lipid bilayer originating from the plasma membrane encasing a cargo containing genetic material, proteins, and lipids, thereby acting as important mediators of intercellular communication (Lawson et al., 2017).

Therefore, it is possible that the respective *N*-glycosylation site is a critical determinant in trafficking of LCN2 to exosomes. In line with this assumption, first evidence for the occurrence of LCN2 in exosomes was demonstrated in urine samples in which the cellular fraction contained lower levels of LCN2 compared with the exosomal fraction (Alvarez et al., 2013).

In the present study, we addressed the question if *N*-glycosylation of LCN2 is necessary for proper secretion or exosome targeting. The inhibition of *N*-glycosylation by tunicamycin in primary hepatocytes resulted in a slimed core protein that retained its capacity to become secreted and recruited to the exosome fraction. Moreover, thapsigargin leading to endoplasmic reticulum Ca^2+^ depletion provoking unfolded protein response failed to inhibit LCN2 expression, *N*-glycosylation or secretion. Therefore, we conclude that the impact of tunicamycin in *N*-glycosylation of LCN2 is linked to inhibition of the GlcNac phosphotransferase, while *N*-linked glycosylation is not necessary for LCN2 exosome cargo recruitment.

## Materials and methods

### Sequence alignment

The protein sequences of LCN2 from human (GenBank:AAH33089.1), rhesus macaque (GenBank:EHH23781.1), Chinese hamster (NCBI Reference Sequence: XP_003512308.2), mouse (GenBank:EDL08545.1), rat (GenBank:AAH89053.1), cheetah (NCBI Reference Sequence:XP_014917872.1), cattle (GenBank:DAA24266.1) horse (NCBI Reference Sequence:XP_005605876.1), and Malayan Pangolin (NCBI Reference Sequence:XP_017497319.1) were aligned using the default settings of Clustal Omega (Sievers et al., 2011) *via* a web interface^2^.

### Signal peptide prediction

The prediction of signal peptide cleavage sites were done with the SignalP 4.1 algorithm (Petersen et al., 2011) *via* a web resource^3^.

### *N*-glycosylation prediction

The primary sequences of human, mouse, and cheetah LCN2 proteins were analyzed for potential *N*-glycosylation site using the NetNGlyc *N*-Glycosylation site predictor with the default parameter settings^4^.

### Hydrophobic cluster analysis

The hydrophobic cluster analysis of the LCN2 protein from different species was done with a web resource for structural Bioinformatics using the default settings^5^.

### Isolation and culturing of primary hepatocytes

Primary hepatocytes from livers of 6–11 week old male wild type or LCN2 deficient mice (Berger et al., 2006) were isolated through a two-step *in situ* collagenase perfusion method following standard procedures (Seglen, 1976). The cells were seeded at a density of 5 × 10^6^ cells/100 mm or 1.4 × 10^6^ cells/60 mm collagen-coated culture dishes and cultivated in HepatoZYME-SFM medium (Thermo Fisher Scientific, Schwerte, Germany). The protocol used for hepatocyte isolation was approved by the respective authority which is the *Landesamt für Naturschutz, Umwelt und Verbraucherschutz Nordrhein-Westfalen* (LANUV) located in Recklinghausen, Germany. For details, see: https://www.lanuv.nrw.de.

### Culturing and differentiation of immortalized cell lines

#### HL-60/dHL-60

Immortalized human promyelocytic leukemia cell line HL-60 was originally derived from peripheral blood of a 36-year-old female patient suffering from acute promyelocytic leukemia (Collins et al., 1977; Gallagher et al., 1979). The cells were routinely grown in RPMI 1640 medium supplemented with 10% heat-inactivated (30 min, 56°C) fetal calf serum (FCS), 100 U/mL penicillin, 100 μg/mL streptomycin, and 2 mM L-glutamine. For the depicted experiments, cells were adapted to advanced DMEM/F12 medium, supplemented with 10% heat-inactivated FCS, 100 U/mL penicillin, 100 μg/mL streptomycin, and 2.5 mM L-glutamine. The cells were stimulated to differentiate into granulocytic cells in advanced DMEM/F12 medium (+ 10% heat-inactivated FCS, 100 U/mL penicillin, 100 μg/mL streptomycin, and 2.5 mM L-glutamine) by addition of 1 μM all-trans retinoic acid (ATRA, Sigma-Aldrich, Taufkirchen, Germany) and 1.25% DMSO (Fluka, Sigma-Aldrich). In agreement with previous reports, the differentiation process forming dHL-60 cells required ~4 days under these conditions (Le Cabec et al., 1997). The stimulation experiments (24 h) were done in cells adapted to advanced DMEM/F12 without FCS and the use of 200 ng/mL LPS (from *Salmonella typhimurium*, Sigma-Aldrich) and/or 50 or 100 ng/mL tunicamycin from *Streptomyces* sp. (#T7765, Sigma-Aldrich).

#### NB4/dNB4

The cell line NB4 was established from the bone marrow of a 23-year-old female patient with acute promyelocytic leukemia (Lanotte et al., 1991; Duprez et al., 1992). The cells were routinely grown in RPMI 1640 medium supplemented with 10% heat-inactivated FCS, 100 U/mL penicillin, 100 μg/mL streptomycin, and 2 mM L-glutamine. Experiments and stimulation experiments were done in cells adapted to advanced DMEM/F12 medium under the same conditions described for HL-60 (see section HL-60/dHL-60). For differentiation into granulocytic cells (dNB4), the medium was supplemented with 1 μM ATRA and 0.4% DMSO for 4–5 days. LPS and/or tunicamycin stimulation was done as described above (HL-60/dHL-60).

#### PC-3

This epithelial human prostate cell line (ATCC® CRL-1435™) established from a metastatic site of bone of a 62-year-old male patient suffering from grade IV adenocarcinoma (Kaighn et al., 1979) was obtained from LGC Standards GmbH (Wesel, Germany) and routinely cultured in DMEM supplemented with 10% FCS, 100 U/mL penicillin, 100 μg/mL streptomycin, and 2 mM L-glutamine. For the experiments, the cells were adapted to advanced DMEM/F12 (+ 10 % FCS, 100 U/mL penicillin, 100 μg/mL streptomycin, and 2.5 mM glutamine). Stimulation with 2.5 ng/mL recombinant human IL-1β (IL-1β, Miltenyi Biotech, Bergisch Gladbach, Germany) and/or 0.5 μg/mL tunicamycin was done for 36 h in advanced DMEM/F12 without FCS.

#### A549

This human epithelial carcinoma lung cell line originated from an explant culture derived from a 58-year-old male patient with solid lung carcinoma (Giard et al., 1973). The cells were routinely grown in DMEM supplemented with 10% FCS, 100 U/mL penicillin, 100 μg/mL streptomycin, and 2 mM L-glutamine. All experiments and stimulation experiments were done in advanced DMEM/F12 under conditions described for PC-3 cells (see PC-3).

#### HepG2

In some Western blot experiments depicted, HepG2 cells stimulated with IL-1β were taken as a positive control for LCN2 expression. This human hepatoma derived cell line isolated from liver biopsies of a child with hepatocellular carcinoma (Knowles et al., 1980). When stimulated with IL-1β, the expression of LCN2 is strongly induced (Borkham-Kamphorst et al., 2011).

#### TW60

The murine hepatoma cell line TW60 was generated in the laboratory of Christian Liedtke (Department of Internal Medicine III, RWTH Aachen University) as described before (Boaru et al., 2015). In brief, this tumorigenic cell line was isolated from hepatocellular carcinoma nodules developed in male mice on C57BL/6 genetic background 40 weeks after single intraperitoneal injection of 25 mg diethylnitrosamine/kg body weight. Cell were routinely grown in DMEM containing 1% non-essential amino acids, 1 mM sodium pyruvate, 2 mM L-glutamine, 1% Penicillin/Streptomycin, and 10% FCS. The stimulation experiments were done in advanced DMEM/F12 under conditions described for PC-3 cells (see PC-3).

### Adenoviral infection

Primary hepatocytes were infected in HepatoZYME-SFM medium with 2.0 × 10^8^ adenoviral particles/mL for 4.5 h. Subsequently, the medium was replaced with new media and cultured for an additional 24 h period. Thereafter, the medium was replaced with media containing tunicamycin at a concentration of 0.5 μg/ml. Twenty-four hours later, the conditioned medium was harvested for isolation of exosomes. Construction, amplification, and purification of AdEasy1-CMV-mLCN2 were done as reported previously (Asimakopoulou et al., 2014).

### Tunicamycin and thapsigargin treatment

One or two days after initial plating of primary hepatocytes, the medium was replaced with fresh medium containing 1–10 μg/mL tunicamycin or 0.025–0.2 μg/mL thapsigargin (#T9033, Sigma-Aldrich) and cultured for additional 24 h. Thereafter, the conditioned cell culture media were harvested and cell protein extracts prepared for Western blot analysis. Cells cultured for the same time without tunicamycin or thapsigargin or in the presence of vehicle (dimethyl sulfoxide, DMSO) were taken as controls.

### Liver injury models

All animal liver specimens used in immunohistochemistry were generated in our laboratory in previous experiments. Therefore, no additional suffering to animals was caused in this study. The former experiments were approved by the LANUV (see also comment in Isolation and Culturing of Primary Hepatocytes).

#### Lipopolysaccharide injection

The application of lipopolysaccharide (LPS) was performed following a well-standardized protocol previously published (Hamesch et al., 2015). In brief, animals received 2.5 mg/kg body weight LPS and were sacrificed 6 h thereafter. Control mice received a same volume (100 μl) of a normal saline solution (NSS).

#### Concanavalin A treatment

A detailed protocol for the application of Concanavalin A (Con A) including preparation of the Con A working solution was previously published (Heymann et al., 2015). Mice received intravenous injection of 25 mg/kg body weight Con A and were sacrificed 8 h thereafter. Livers from mice injected with NSS served as control.

#### Bile duct ligation

Induction of experimental obstructive cholestasis in mice was induced by bile duct ligation (BDL), essentially following protocols outlined elsewhere (Tag et al., 2015a,b). Animals were sacrificed 4 weeks after the surgery and sham-operated mice served as controls.

#### Carbon tetrachloride injection

Repeated administration of carbon tetrachloride (CCl_4_) in mice was performed as outlined in detail elsewhere (Scholten et al., 2015). In brief, chronic intoxication with CCl_4_ was done by intraperitoneal injection of 0.6 μL/g body weight diluted in corn oil. The injections were performed twice per week for 8 consecutive weeks. Mice receiving equal volumes of corn oil alone served as controls.

### SDS-page and western blot analysis

Conditioned cell media were collected and cell lysates prepared in RIPA buffer containing 20 mM Tris-HCl (pH 7.2), 150 mM NaCl, 2% (w/v) NP-40, 0.1% (w/v) SDS, 0.5% (w/v) sodium deoxycholate and the Complete™-mixture of proteinase inhibitors (Roche Diagnostics, Mannheim, Germany). The protein concentration of each sample was determined using the DC protein assay (Bio-Rad Laboratories GmbH, Munich, Germany). Equal protein amounts of cellular extracts (30 μg) or volumes of conditioned cell culture media (max. 25 μl) were diluted with Nu-PAGE™ LDS electrophoresis sample buffer supplemented with DTT as reducing agent, heated at 95°C for 10 min, and separated in 4–12% Bis-Tris gradient gels, using MOPS or MES running buffers (all from Invitrogen, Thermo Fisher Scientific). Proteins were electroblotted onto nitrocellulose membranes (Schleicher & Schuell BioScience, Dassel, Germany), and equal loading was documented in Ponceau S stain. Subsequently, non-specific binding sites were blocked in TBS containing 5% (w/v) non-fat milk powder and probed with primary antibodies that were diluted in 2.5% (w/v) non-fat milk powder in Tris-buffered saline. Primary antibodies used were: LCN2 (#AF3508, R&D Systems, Bio-Techne, Wiesbaden-Nordenstadt, Germany), IRE1α (#3294, Cell Signaling, Darmstadt, Germany), peIF2α (#3597, Cell Signaling), eIF2α (#9722, Cell Signaling), BIP (#3177, Cell Signaling), CHOP (#2895 or #5554, Cell Signaling), pp65 (#3033, Cell Signaling), p65 (sc-8008, Santa Cruz Biotech., Santa Cruz, CA, USA^6^), CD81 (B-11) (sc-166029, Santa Cruz), Alix (1A12) (sc-53540, Santa Cruz), p21 (#556430, BD Pharmingen, Heidelberg, Germany), MPO (#HP9048, HycultBiotech, Beutelsbach, Germany), and GAPDH (sc-32233, Santa Cruz Biotech.). The primary antibodies were visualized using horseradish peroxidase (HRP)-conjugated anti-mouse-, anti-rabbit-, or anti-goat IgG (all from Santa Cruz Biotech.) and the SuperSignal chemiluminescent substrate (Pierce, Bonn, Germany).

### Method for isolation of exosomes from conditioned media

Isolation of exosomes from cell culture conditioned media of hepatocytes was carried out following an ultracentrifugation protocol as described by others considering small modifications (Lobb et al., 2015). Briefly, the conditioned media was harvested from primary murine hepatocytes and centrifuged using a Heraeus Sepatech refrigerated centrifuge at 600 g at 4°C in a BS 4402/A rotor for 15 min to remove detached cells. Supernatant was collected and again centrifuged at 3,200 g at 4°C for 30 min to remove cell fragments. The second supernatant was collected and filtered slowly through 0.22 μm sterile syringe filters (#431219, Corning GmbH, Kaiserslautern, Germany) to remove contaminating apoptotic bodies, microvesicles and cell debris. Cleared supernatant was then centrifuged in a Beckman Optima^TM^ L-70K Ultracentrifuge equipped with a SW 40 Ti rotor at 100,000 g (29,500 rpm; RCF_avg_ 109,895; RCF_max_ 154,779; k-factor: 252,5) at 4°C for 70 min to pellet exosomes. The supernatant was carefully removed, and crude exosome-containing pellets were washed in ice-cold 200 mM 4-(2-hydroxyethyl)-1-piperazineethanesulfonic acid (HEPES) buffer (pH 7.0) and pooled. A second round of ultracentrifugation under the same conditions was carried out, and the resulting exosome pellet resuspended in 200 μL of 200 mM HEPES buffer (pH 7.0) for electronmicroscopic analysis and NTA measurements, or in 200 μl of 50 mM HEPES buffer (pH 7.0) for Western blot analysis.

### Isolation of exosomes from established cell lines

For isolation of exosomes from immortalized cell lines, the cells were grown in DMEM, RPMI 1640, or advanced DMEM/F12 supplemented with 10% heat inactivated or normal FCS, 100 U/mL penicillin, 100 μg/mL streptomycin, and 2.5 or 2 mM L-glutamine. TW60, HL-60, dHL-60, NB4, dNB4, A549, and PC-3 cells were stimulated for 24 h in serum free advanced DMEM/F12 medium with LPS or IL-1β, and/or tunicamycin. At the end of the incubation, cell culture media were taken for preparation of exosomes following the protocol given above (see Method for Isolation of Exosomes from Conditioned Media).

### Sizing and concentration measurement of exosome suspension by nanoparticle tracking analysis

Particle size and concentration of exosome preparations were measured using the NanoSight NS300 instrument (Malvern Instruments Limited, Malvern, Worcestershire, UK) allowing particle concentration to be visualized and measured at 10^6^-10^9^ particles per mL in the 10 nm–2,000 nm diameter range in liquid suspension. The high resolution size distributions on a particle-by-particle basis are counted in real-time and the quantities of purified exosome suspensions of each five individual measurements were combined and visualized with the NanoSight NS300 nanoparticle tracking analysis (NTA) v 3.00 software (Malvern Instruments Limited). For details see (Malvern Application note).

### Transmission electron microscopy of exosomes

Unfixed isolated exosomes in 200 mM HEPES buffer (pH 7.0) were allowed to adsorb on glow discharged Formvar/carbon-coated nickel grids (Maxtaform, 200 mesh, Plano, Wetzlar, Germany) for 3 min. Samples on grids were contrasted by placing on a drop of ready-made pre-packed 0.5% uranyl acetate in aqua dest. (Science Services GmbH, Munich, Germany). After air drying, samples were examined using a TEM LEO 906 (Carl Zeiss, Oberkochen, Germany), operating at an acceleration voltage of 60 kV. Representative images of exosomes were taken over a magnification range from 60,000 to 359,700x.

### Immunohistochemistry

Liver tissue sections obtained from mice treated with LPS, Con A, CCl_4_, or subjected to BDL were prepared for immunohistochemical analysis as described previously (Borkham-Kamphorst et al., 2008). Staining for LCN2 and neutrophil marker MPO was performed under conditions given elsewhere (Borkham-Kamphorst et al., 2013; Asimakopoulou et al., 2016a).

## Results and discussion

### Hepatic expression of LCN2

**Figure 1:**
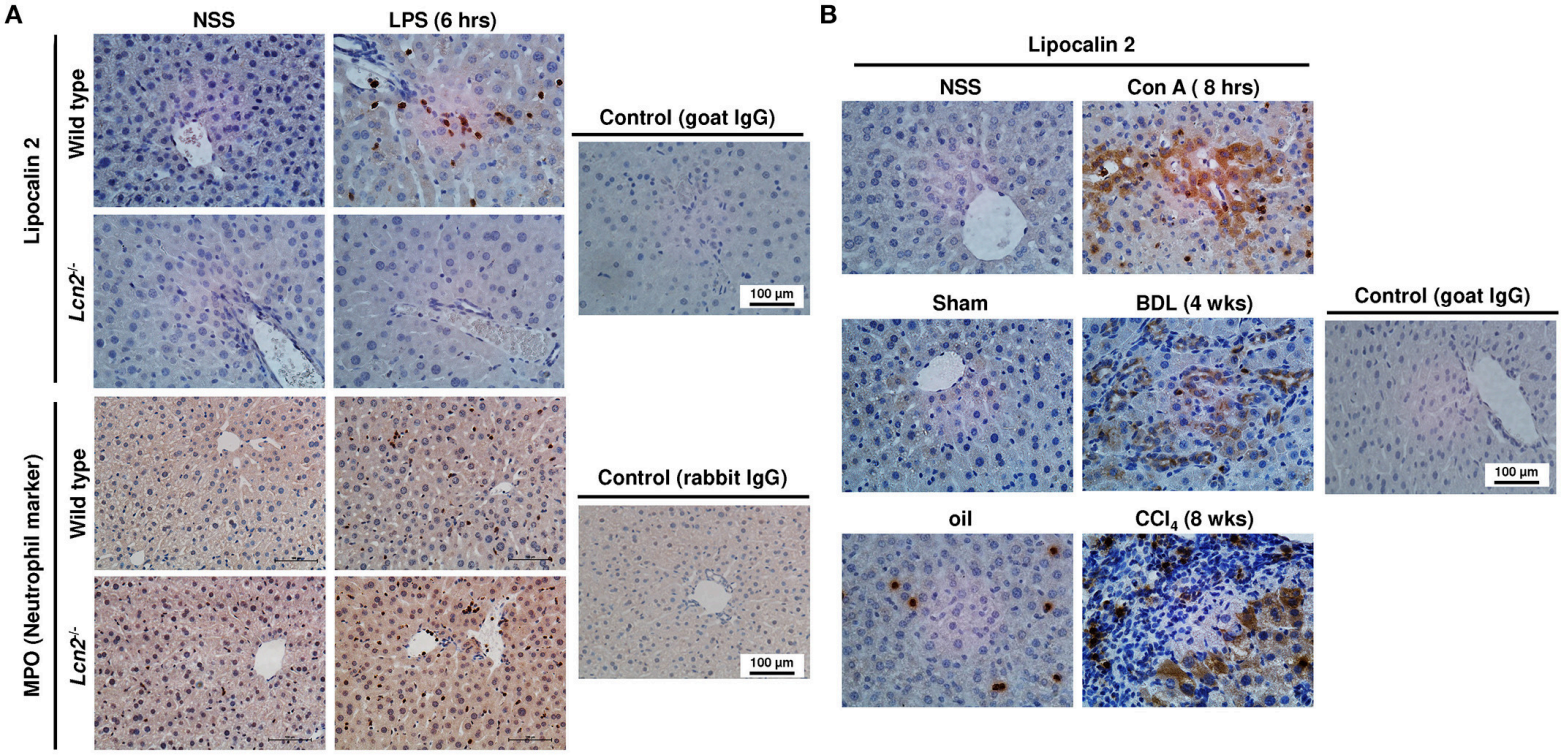
Immunohistochemistry of LCN2 expression in liver injury. **(A)** Sections were prepared from livers obtained from animals 6 h after LPS injection and stained for LCN2 and MPO. Liver sections obtained from normal standard saline (NSS)-injected animals and from *Lcn2* deficient mice served as controls. **(B)** Liver sections from animals treated with Con A for 8 h **(upper)**, subjected to bile duct ligation (BDL) for 4 weeks **(middle)**, or from animals receiving CCl_4_ for 8 weeks **(lower)** were immunostained for LCN2. Control sections from NSS-treated, sham-operated, or oil-injected mice were taken as controls. In addition, staining with unspecific goat or rabbit control immune sera served as controls.

There are many mediators and pathways contributing to elevated expression of LCN2 in a wide variety of cells, including primary inflammatory cells, macrophage cell lines, epithelial cells, cancer cell lines, and many others (Asimakopoulou et al., 2016b). In regard to liver, we have previously shown that the LCN2 expression dramatically increases *in vivo* in response to damage, inflammation, and in primary isolated hepatocytes cultured for prolonged times (Borkham-Kamphorst et al., 2011, 2013; Labbus et al., 2013; Asimakopoulou et al., 2016a). When comparing acute and chronic models of hepatic injury, it is evident that in principle different resident and infiltrating cells are capable to express LCN2 in the damaged tissue (Figure 1). In livers obtained from mice intraperitoneally injected with Lipopolysaccharide (LPS) for 6 h, neutrophils significantly increasing in number are most prominently immunopositive for LCN2 (Figure 1A). After intravenous application of the plant lectin Concanavalin A (Con A), the resulting liver damage is associated with a strong expression of LCN2 in bile duct epithelial cells, hepatocytes, and a concomitant expression of LCN2 in infiltrating immune cells (Figure 1B). Similarly, after BDL for 4 weeks, LCN2 immunopositive cells are located in the portal tract and in the parenchyma. Moreover, expression of LCN2 in livers of mice subjected to repeated CCl_4_ injections for 8 weeks is majorly found in areas of damaged parenchymal cells (i.e., hepatocytes) and in infiltrating immune cells present within the inflamed tissue. Interestingly, also the peritoneal injection of corn oil for 8 weeks alone provoked a significant fraction of LCN2 positive neutrophils. However, their overall quantity was lower when compared to tissue obtained from animals receiving CCl_4_. In addition, the application of corn oil was not associated with elevated expression of LCN2 in hepatocytes. All these data confirm our previous results showing a rapid and well-sustained induction of LCN2 in hepatic injury (Borkham-Kamphorst et al., 2011). Based on the fact that hepatocytes are “metabolic overachievers” representing the predominant cell type in liver with ~135 million cells per gram of murine liver tissue occupying more than 80% of the liver volume (Sohlenius-Sternbeck, 2006; Gao et al., 2008), it is reasonable to suggest hepatocytes as one (most likely the major) source of hepatic LCN2 expression. This assumption supports previous findings suggesting LCN2 as an acute phase protein, primarily expressed and secreted by hepatocytes during the acute phase of inflammation (Liu and Nilsen-Hamilton, 1995).

### *N*-glycosylation of LCN2

*N*-glycosylation in eukaryotic organisms is generally mediated through a membrane protein complex (i.e., the oligosaccharyltransferase) located on the luminal face of the endoplasmic reticulum to catalyze a transfer of a 14-sugar oligosaccharide to individual Asn residues located in specific acceptor peptide sequences (Mellquist et al., 1998). These sequons are comprised of an Asn-X-(Ser/Thr) or less frequently of an Asn-X-Cys tripeptide where X can be any amino acid except for proline (Moremen et al., 2012). A functional *N*-glycosylation site in human LCN2 susceptible to treatment with *N*-glycanase was already identified in the original study reporting LCN2 identification and purification (Kjeldsen et al., 1993). Consequently, digestion of murine LCN2 with *N*-glycosidase F produced a protein with a reduced molecular mass (Chu et al., 1996). Although *N*-glycosylation of LCN2 was not analyzed in other species, it is obvious that the respective site is evolutionarily conserved (Supplementary Figure 1A) suggesting that the NVTS motif (NATS in cheetah) is of fundamental importance for LCN2 post-translational modification and function. Only the sequences of mouse and cheetah showed an additional predicted *N*-glycosylation site (Supplementary Figure 1B) located in the close proximity of the evolutionarily conserved sequon. Recent work has demonstrated that *N*-glycosylation in the ER is of fundamental importance for glycoprotein folding and for ER-associated degradation of misfolded glycoproteins (ERAD) (Roth and Zuber, 2017). Moreover, *N*-linked glycosylation is critical for protein maturation and used as a quality control signal in the secretory pathway that when altered provoking ER stress and unfolded protein response (Wang et al., 2015). The *N*-glycosylation site of LCN2 is located in front of a cysteine residue (Cys 96 in human LCN2), proposed to be necessary to form LCN2 homodimers, oligomers, heterodimers, or intramolecular disulfide bounds (Kjeldsen et al., 1993; Nickolas et al., 2012; Shukla et al., 2017).

### Inhibition of *N*-glycosylation

**Figure 2:**
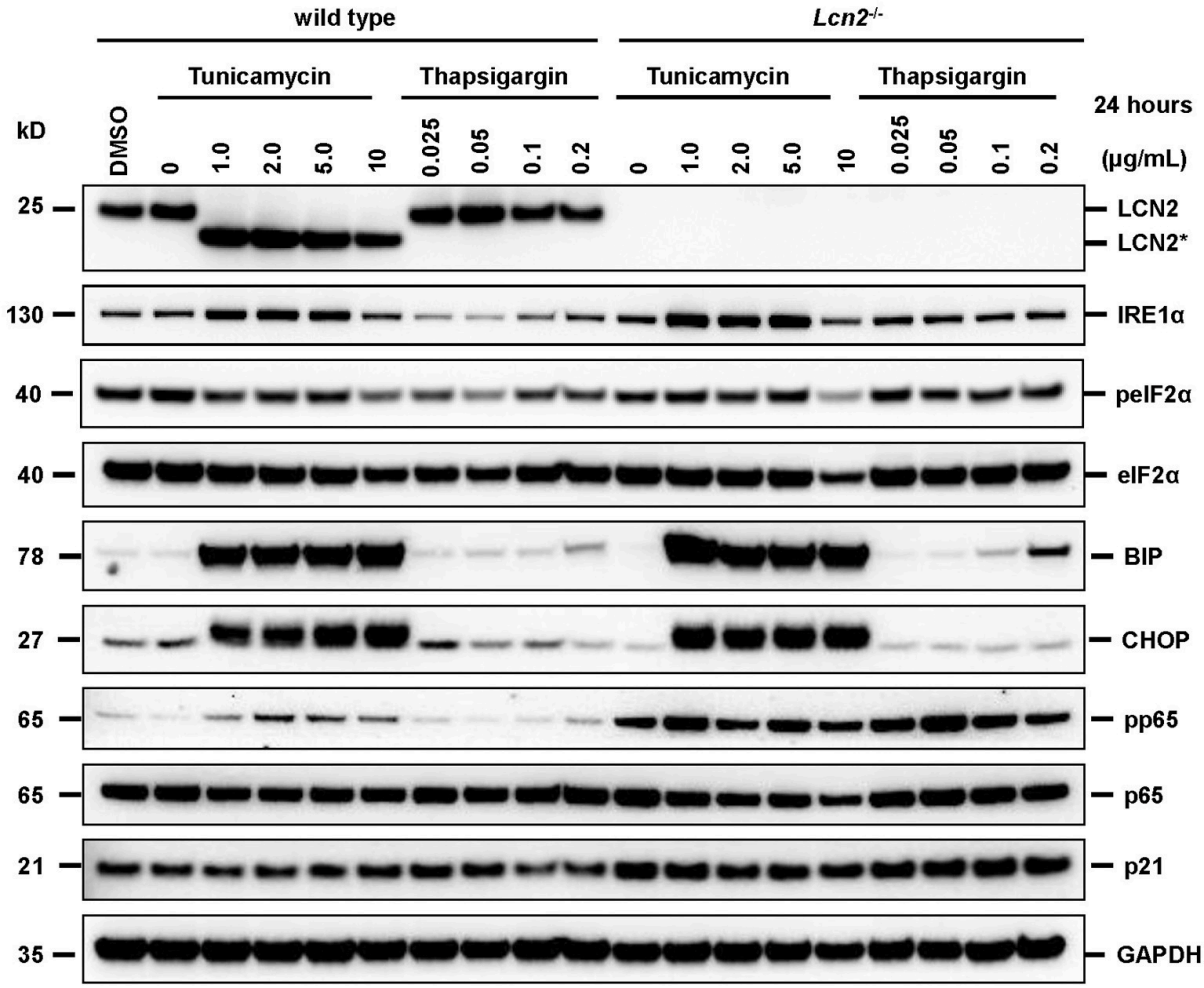
Western blot analysis of cell extracts. Primary murine hepatocytes isolated from wild type or *Lcn2* deficient were incubated for 24 h in the presence of indicated concentrations of tunicamycin (a *N*-acetylglucosamine transferase inhibitor) or thapsigargin (a non-competitive inhibitor of the sarco/endoplasmic reticulum Ca^2+^-ATPase and ER stressor). Cell extracts were prepared and subjected to Western blot analysis. The blots were probed with indicated antibodies. Cell extracts from cells without drug treatment and vehicle (DMSO)-treated cells served as controls. The probing with a GAPDH specific antibody was performed to demonstrate equal protein loading in each lane. Please note the reduced size of LCN2 (LCN2^*^) in cells that were treated with tunicamycin.

**Figure 3:**
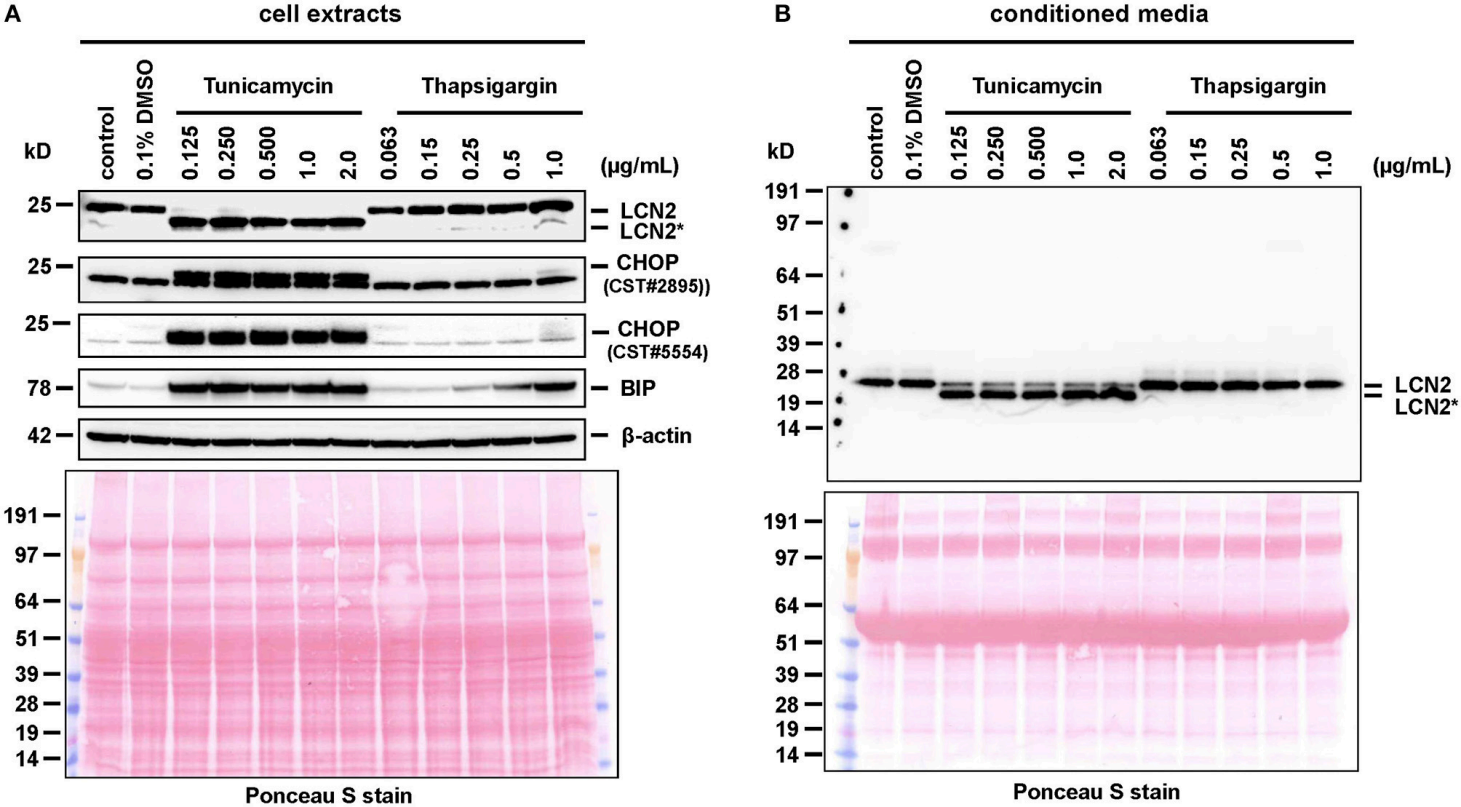
Western blot analysis of cell extracts and conditioned media. **(A)** Cell extracts or **(B)** conditioned media of primary hepatocytes cultured for 24 h in the presence of tunicamycin, thapsigargin, or without any additional supplement were subjected to Western blot analysis and probed for indicated proteins. Please note that the cell extracts were probed for CHOP with two different antibodies (CST #2895 and CST #5554), which identified two protein bands with a preference for the upper band supposed to be CHOP. The Ponceau S stains were taken to document equal protein loading. Please note the lower molecular weight of LCN2^*^ that is due to lack of *N*-glycosylation.

We found that the glycosylation of LCN2 was significantly inhibited in cultured hepatocytes by tunicamycin (b). In the Western blot analysis, the respective treatment resulted in a partial reduction in the apparent molecular mass from 25 to 22 kDa (the calculated molecular mass of unglycosylated murine LCN2 without secretory signal is 20.87 kDa) (Figure 2). Since we first speculated that proper *N*-glycosylation in LCN2 is a prerequisite for effective secretion, we thought that the *N*-glycosylation inhibitor tunicamycin should inhibit efficiently LCN2 secretion in cultured hepatocytes. In these cells LCN2 expression increases dramatically during prolonged culturing (Borkham-Kamphorst et al., 2011). However, the treatment with tunicamycin even at lower concentrations had no impact on the secretion of LCN2 suggesting that this post-translational modification is not necessary for proper secretion (Figure 3). Thapsigargin, a non-competitive inhibitor of the ubiquitous sarco/endoplasmic reticulum Ca^2+^-ATPase (SERCA) and ER stressor, failed to interfere with LCN2 glycosylation. The low traces of unglycosylated LCN2 in cell extracts of hepatocytes treated with high concentrations (1 μg/mL) of thapsigargin were mostly likely provoked by dying hepatocytes that have reduced biochemical capacity under the chosen experimental setting. In line with this notion, thapsigargin was previously shown to induce programmed cells death or necrosis in primary rodent hepatocytes and different hepatoma cell lines when applied in similar concentrations (Xie et al., 2002; Sohn et al., 2003; Chae et al., 2004; Li and Holbrook, 2004; Wang et al., 2016).

In addition, this finding indicates that the missing *N*-glycosylation does not provoke LCN2 degradation during processing through the ER and Golgi apparatus. At the same time, the treatment with tunicamycin induced ER stress and activated the unfolded protein response (UPR). After treatment with tunicamycin, we observed elevated expression of the inositol-requiring enzyme 1α (IRE1α) and a strong upregulation of BIP/GRP-78 and CHOP/DDIT3 that indicate ER stress (cf. Figure 2). In addition, this analysis revealed that the general lack of LCN2 in cells originating from LCN2-deficient mouse provokes an increased activity of the NF-κB pathway. This pathway is critically involved in the induction of LCN2 in cultured hepatocytes and other cell entities (Borkham-Kamphorst et al., 2011; Zhao and Stephens, 2013). Possibly, the elevated quantities of activated pp65/RelA and the activation of the NF-κB pathway are mechanisms to compensate the lack of LCN2 or are signs of early cellular damage and initiation of apoptotic processes.

### LCN2 is partially targeted to exosomes in primary hepatocytes

Glycosylation is the most common post-translational modification in eukaryotic cells crucially involved in protein trafficking to specific membranes. In particular, *N*-glycans are critical determinants in trafficking and sorting of apical membrane proteins in epithelia (Vagin et al., 2009). There is also strong evidence that *N*-linked glycosylation directs glycoprotein sorting into exosomes (Liang et al., 2014). To clarify whether the glycosylation state of LCN2 impacts exosome targeting, we next comparatively determined the content of LCN2 in exosomes isolated from conditioned media of primary murine hepatocytes triggered to produce large quantities of LCN2 by infection with AdEasy1-CMV-mLCN2 either in the presence or absence of tunicamycin. The exosomes from respective conditioned media were isolated by differential centrifugation and characterized by electronmicroscopic analysis and sized by NTA. In NTA analysis, the vesicles are visualized by light scattering using a light microscope. During a defined time course, a video is taken and the NTA software tracks the Brownian motion of individual vesicles and calculates their size (Dragovic et al., 2011). In regard to size analyses of extracellular vesicles, the method was shown to have good repeatability and precision in consecutive measurements. In particular, previous studies determined the intra-assay coefficient of variation to 1–5% for size measurements when using the NanoSight NS500 instrument that we used in our study (Vestad et al., 2017). In line with these reports, the NTA measurements in our hands had a good performance, allowing reliable size measurements of high numbers of particles (Supplementary Videos 1, 2).

**Figure 4:**
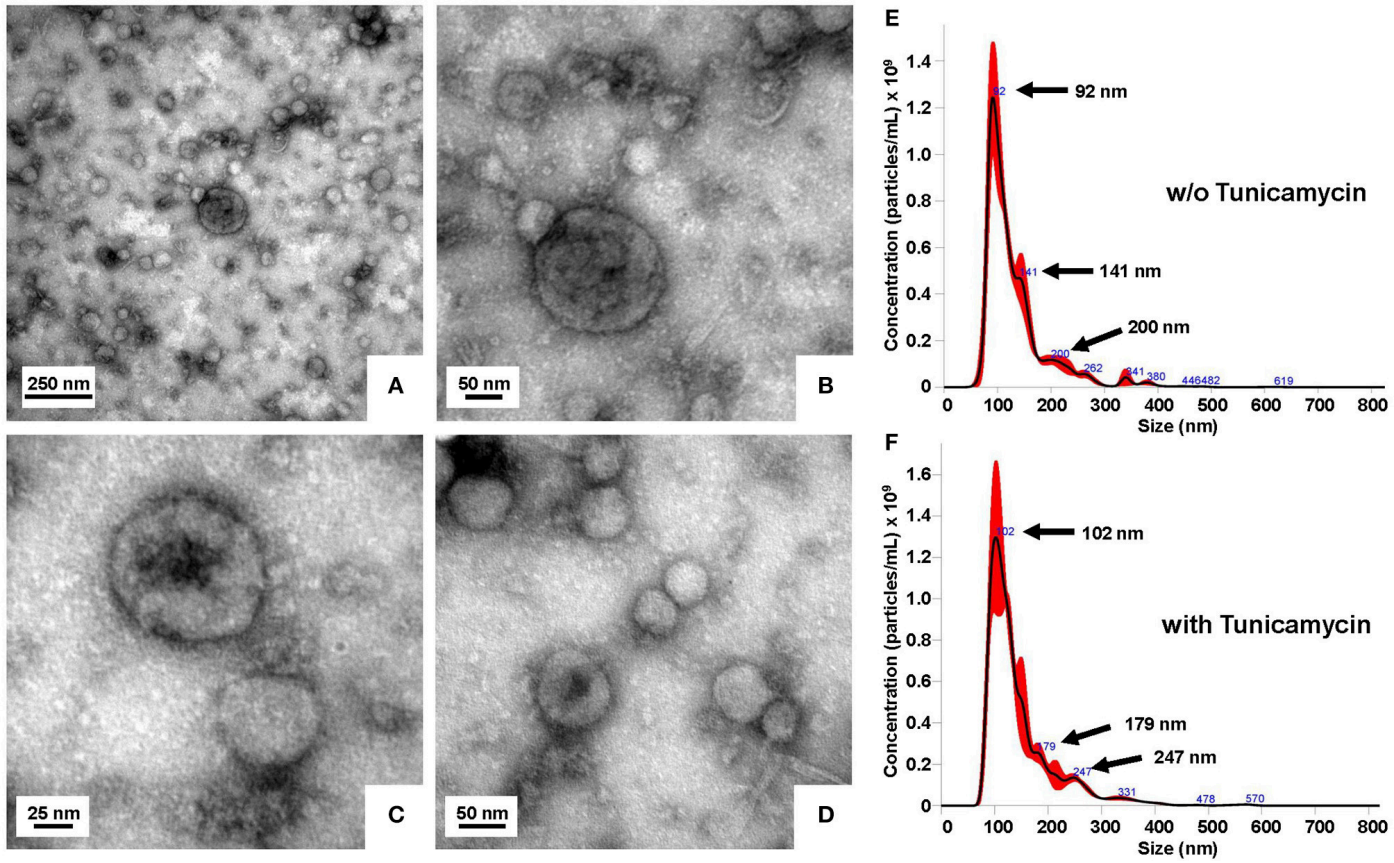
Characterization of isolated exosomes isolated from conditioned medium. **(A–D)** Representative transmission electron microscopic pictures of isolated exosomes negatively stained with 0.5% uranyl acetate. Images were taken at original magnifications of **(A)** 60,000, **(B)** 167,000, **(C)** 359,700, and **(D)** 215,600, respectively. **(E,F)** Exosomes were isolated from conditioned media of Ad5-CMV-mLCN2-infected primary hepatocytes cultured for 24 h in the presence of **(E)** 0.01% DMSO (control) or **(F)** 0.5 μg/mL tunicamycin. Particle size distribution and calculated concentrations as determined by nanoparticle tracking analysis of purified exosome suspensions are depicted.

**Figure 5:**
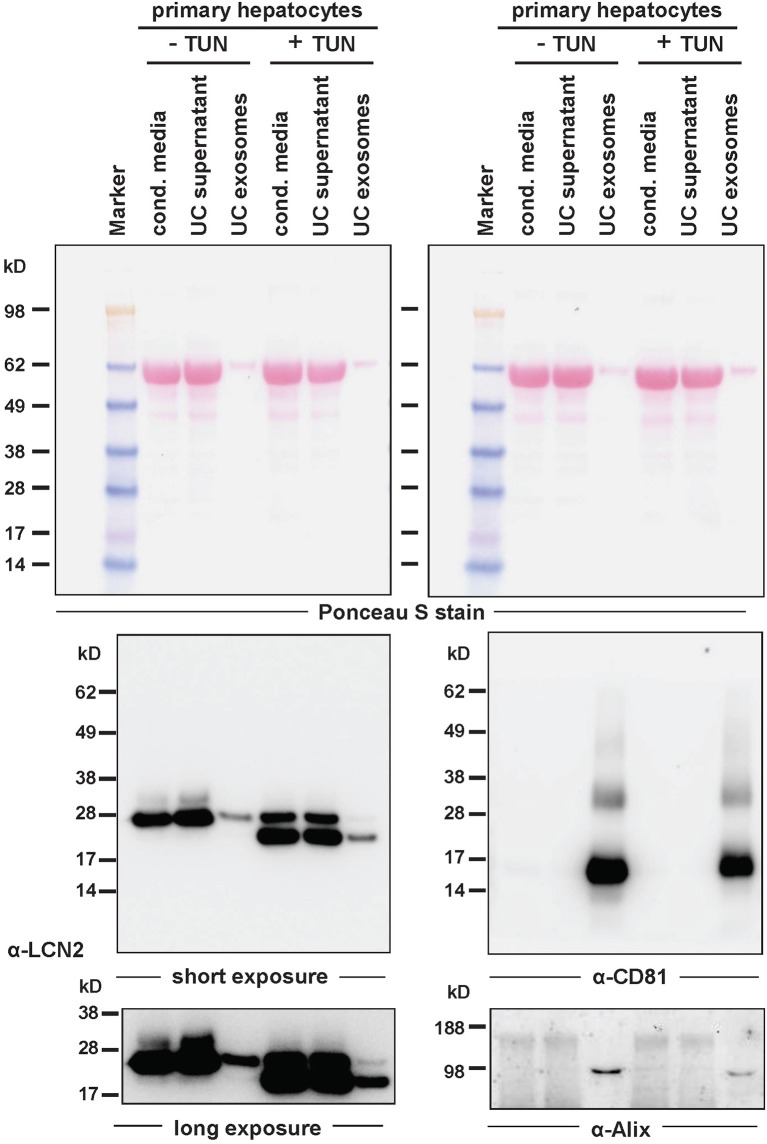
Targeting of LCN2 into exosomes of primary hepatocytes. Exosomes were prepared from conditioned media isolated from AdEasy1-CMV-mLCN2-infected primary murine hepatocytes treated with tunicamycin (+TUN) or left untreated (–TUN). Fractions of conditioned media as well as supernatant (UC supernatant) and exosome pellet (UC exosomes) after ultracentrifugation were then tested in Western blot for expression of LCN2 **(left)** or exosome markers CD81 and Alix **(right)**. Ponceau S stain served to document equal transfer of proteins from the SDS gel to the membrane, while short and long exposure times for the LCN2 specific Western blot are depicted for better estimation of LCN2 quantities targeted to the exosome fraction.

The purified exosomes had the typical size distribution peaking around 100 nm (Figure 4). Subsequent Western blot analysis of purified exosome fractions revealed that both the glycosylated and the non-glycosylated LCN2 variants were found at equal levels in the exosome fraction (Figure 5). Notably, compared to other classical exosome markers including CD81 (also known as target of antiproliferative antibody 1, TAPA1) and ALG-2 interacting protein X (Alix, or Programmed cell death 6-interacting protein, PDCD6IP), the overall quantities of LCN2 included in exosomes was significant lower than the freely secreted fraction. Interestingly, most of the exosomes isolated from tunicamycin-treated hepatocytes were somewhat greater in size than those isolated from vehicle (DMSO)-treated cells. While most particles from tunicamycin-treated cells had a size around 102 nm, the mean vesicle size of control exosomes was 92 nm. Similar shifts in mean vesicles sizes as a consequence of tunicamycin-induced ER stress were recently reported in BeWo choriocarcinoma cells (Collett et al., 2018).

**Figure 6:**
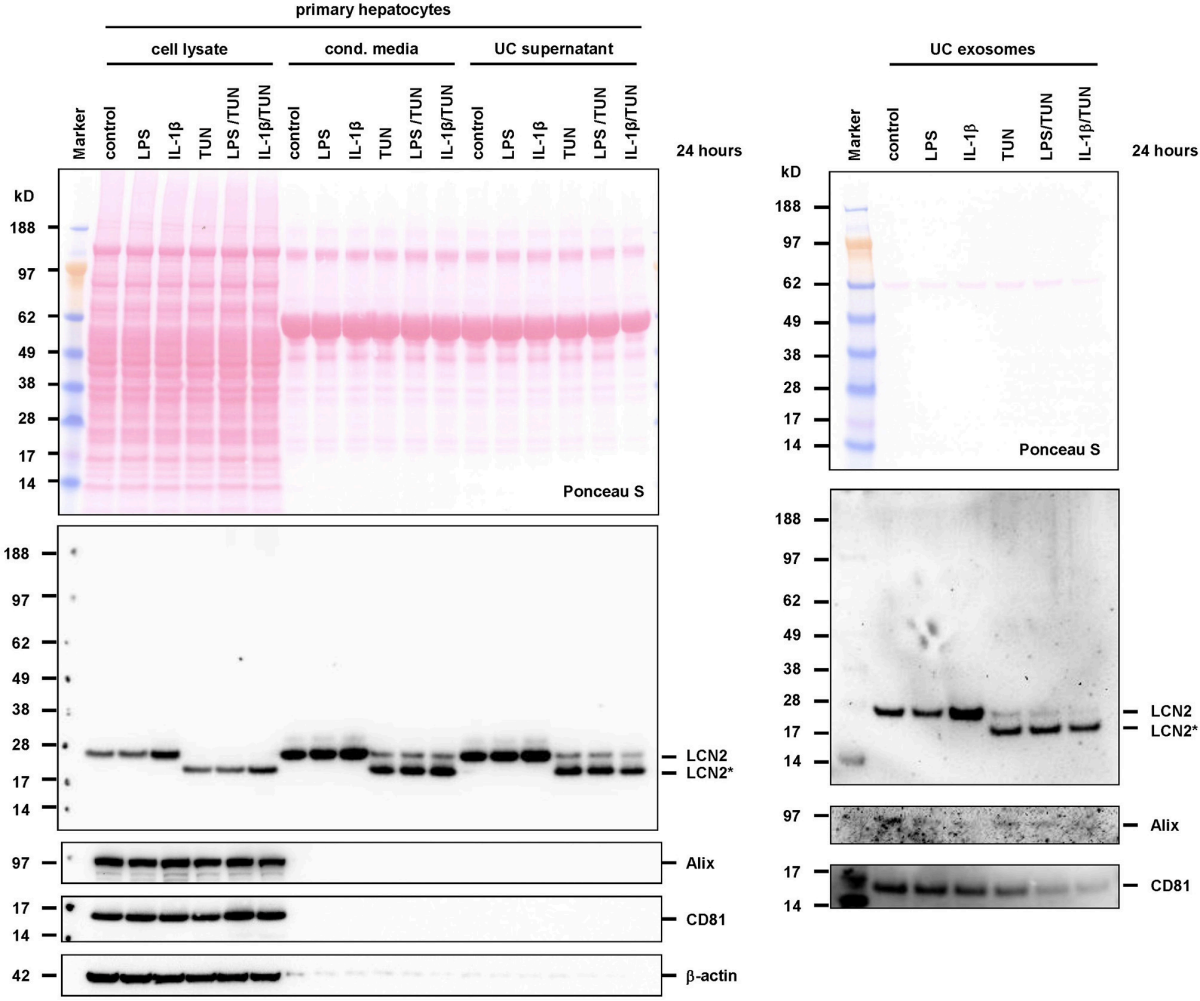
Targeting of LCN2 into exosomes of stimulated primary hepatocytes. Primary murine hepatocytes were stimulated for 24 h with 400 ng/mL LPS or 2.5 ng/mL IL-1β in the presence or absence of 0.5 μg/ml tunicamycin. Protein cell extracts and conditioned media were harvested and exosomes isolated. The different fractions were then tested in Western blot for LCN2 expression or exosome markers CD81 and Alix. Ponceau S stain served to document equal transfer of proteins from the SDS gel to the membrane. In this set of experiments unstimulated cells served as control. Please note that the stimulation with IL-1β resulted in increase of LCN2 in cell extracts and exosomes.

We next tested if LCN2 secretion and exosome targeting of glycosylated and non-glycosylated forms is also observed under conditions in which cells were stimulated by exogenous stimulus. To do so, we stimulated primary hepatocytes with LPS or IL-1β in the presence or absence of tunicamycin. Although LCN2 expression without further stimulation was already high, the addition of 2.5 ng/mL IL-1β resulted in a further increase of LCN2 expression and secretion (Figure 6). In addition, also in the presence of IL-1β or LPS, both LCN2 forms were partially targeted to the exosomes. The stimulation with 400 ng/mL LPS, however, was not able to further increase LCN2 expression during the 24 h incubation period, while the offset in LCN2 expression after IL-1β treatment once again reflects the high capacity of this cytokine in stimulating LCN2 expression (Borkham-Kamphorst et al., 2011). To unequivocally demonstrate the biological activity of the applied LPS, we in parallel treated the murine hepatoma cell line TW60 with respective agents and analyzed cell extracts and conditioned media for LCN2 expression showing LPS- and IL-1β-induced stimulation and secretion of both LCN2 forms (Supplementary Figure 2).

### Expression of LCN2 in human myeloid cell lines

LCN2 was first identified as a neutrophil gelatinase-associated lipocalin stored in specific granules of human neutrophils (Kjeldsen et al., 1993). Therefore, we next wanted to test if cells of the neutrophilic lineage are capable to target LCN2 to exosomes. However, unlike other cell types, primary neutrophils are extremely fragile and rapidly undergo apoptosis when cultured (Haslett et al., 1994). That's why we decided to perform our next experiments in established human promyeloic cell lines. The cell lines HL-60 and NB4 are two human leukemic cell lines commonly used to examine myeloid differentiation. Neutrophil maturation is induced in these cell lines by culturing in the presence of ATRA and/or DMSO for 4–5 days (Gupta et al., 2014). In addition, tunicamycin at low doses inhibit *N*-linked glycosylation in these cells (Pérez-Sala and Mollinedo, 1995). We were somewhat surprised to notice that LCN2 was not detectable in cell extracts or conditioned media of NB4 cells (Supplementary Figure 3) or HL-60 cells (not shown). Moreover, also differentiated dHL-60 and dNB4 showed no detectable expression of LCN2 in cell extracts or conditioned media from respective cells (Supplementary Figures 4, 5). To rule out that myeloid differentiation was not properly induced, we stained the cell extracts for myeloperoxidase (MPO). This analysis resulted in the detection of the typical MPO protein bands with variable molecular weight resulting from a series of complex translational and posttranslational processes of the 80-kDa protein, which is converted into a 90-kDa apo-proMPO, and subsequent formation of short-lived 74-kDa intermediate that is cleaved into two subunits comprising a 59-kDa heavy (α)-subunit and a 13.5-kDa light (β)-subunit (Hansson et al., 2006; van der Veen et al., 2009). To sum up, although both immortalized cell lines expressed the typical myeloid marker MPO, they lacked LCN2.

### Expression, glycosylation, and exosome targeting in other cell types

After we have discarded our plans to use HL-60 or NB4 for our studies, we searched the literature for cell lines known to express LCN2. Human lung adenocarcinoma A549 cells were shown in independent studies to express LCN2 (Tong et al., 2005; Roudkenar et al., 2008). Moreover, LCN2 expression in these cells is inducible by LPS stimulation *via* the IL-1 pathway (Cowland et al., 2003). Likewise, LCN2 expression was previously determined by Western blotting, qRT-PCR, and immunohistochemistry in the human prostate cell line PC-3 (Tung et al., 2013).

**Figure 7:**
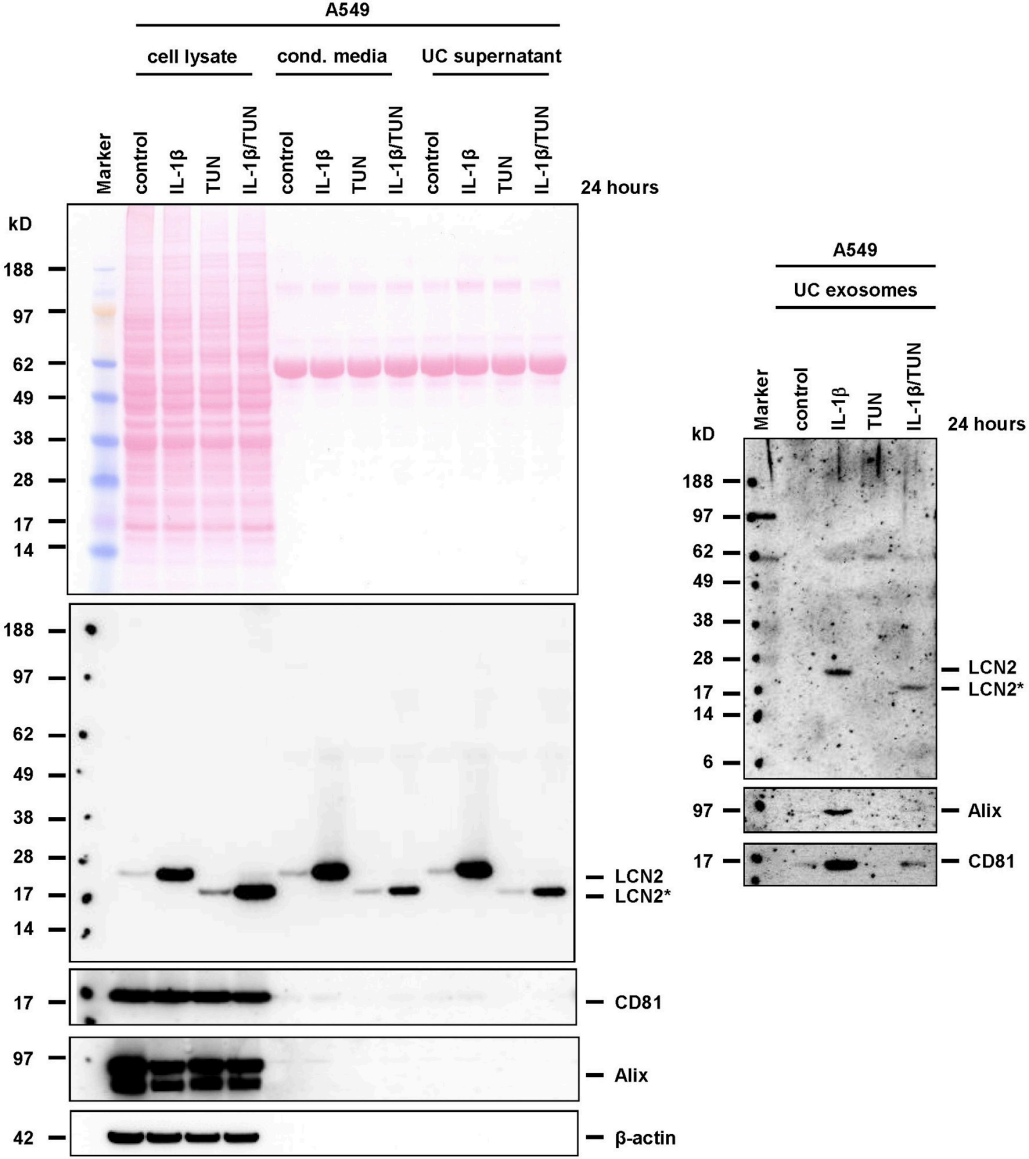
Expression and targeting of LCN2 into exosomes in A549 cells. A549 cells were stimulated with IL-1β, tunicamycin (TUN), IL-1β, and TUN, or left untreated. Cell extracts, conditioned cell culture media, media after ultracentrifugation (UC supernatant), and exosome pellets (UC exosomes) were analyzed for LCN2 expression in Western blot analysis. The blots were reprobed with exosome markers Alix and CD81, while GAPDH expression served as control to demonstrate equal gel loading in cell extract fractions. Please note, the occurrence of two specific Alix protein bands in the A549 cell lysates. Based on provider's information, these bands are found in many human immortalized cell lines including myelogenous leukemia line K-562, and (pro-) monocytic-like lines THP-1 and U-937 when using this mouse monoclonal antibody directed against full length human Alix (Santa Cruz Biotechnology).

**Figure 8:**
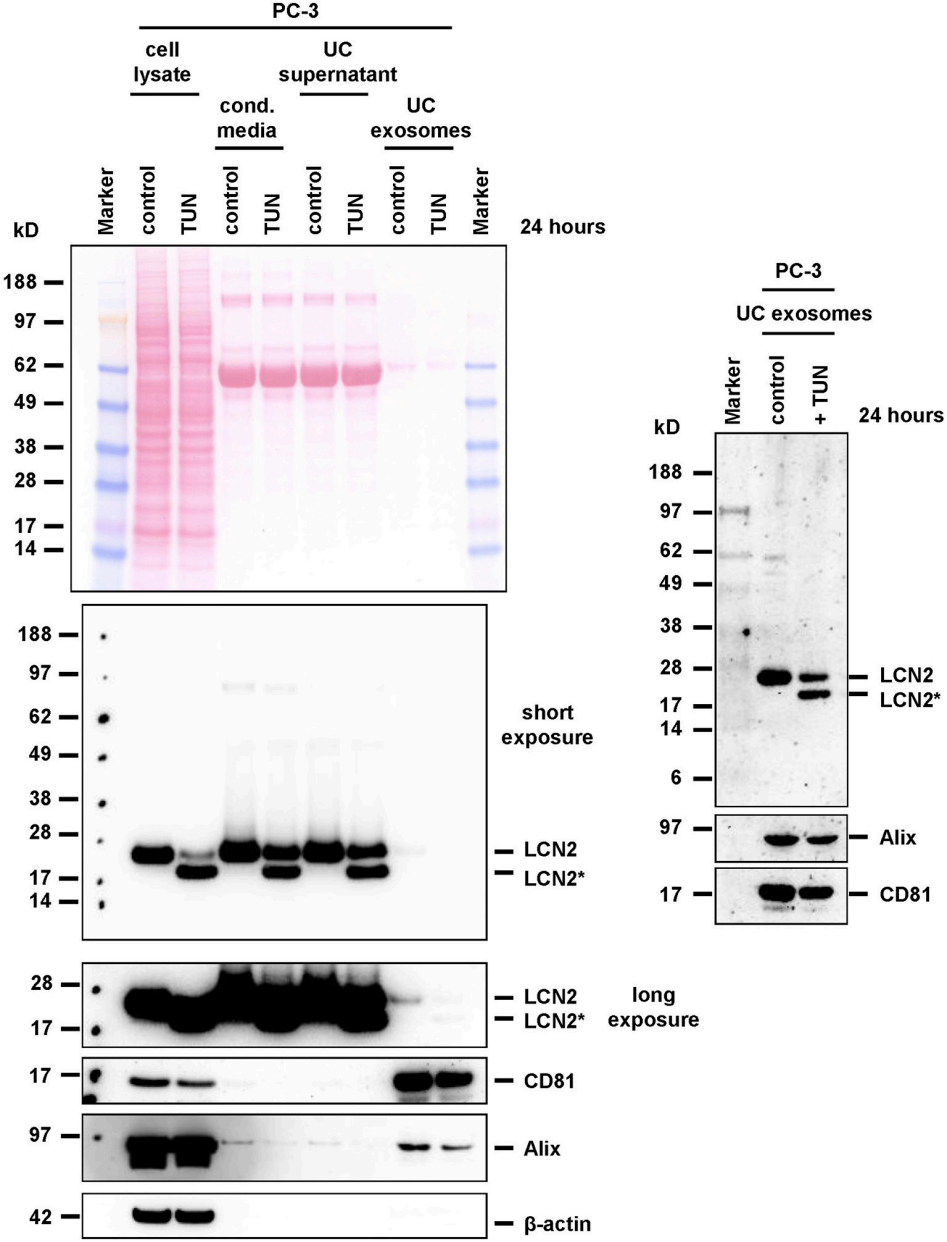
Expression and targeting of LCN2 into exosomes of PC-3 cells. PC-3 cells were treated with tunicamycin (TUN) or left untreated. Cell extracts, conditioned cell culture media, media after ultracentrifugation (UC supernatant), and prepared exosome pellets (UC exosomes) were analyzed for LCN2 expression. The blots were reprobed with exosome markers Alix and CD81, while GAPDH expression served as loading control.

Accordingly, we observed a significant stimulation of LCN2 expression and secretion in A549 cells when we stimulated them with IL-1β (Supplementary Figure 6). When we applied tunicamycin during IL-1β stimulation, the antibiotic was suitable to block *N*-glycosylation. In PC-3, tunicamycin also effectively blocked *N*-glycosylation. However, in these cells the basal high expression of LCN2 was not further triggered by addition of IL-1β, most likely due to the fact PC-3 cells release large quantities of IL-1β, IL-1α (Voss et al., 2010), and IL-6 (Okamoto et al., 1997) leading to autocrine stimulation of LCN2 expression by these cytokines (Borkham-Kamphorst et al., 2011). Although IL-1β-stimulated A549 express only low quantities of LCN2, we were able to demonstrate that both, glycosylated and unglycosylated LCN2 variants, were targeted into exosomes (Figure 7). Likewise, repetition of these experiments in PC-3 cells confirmed that other epithelial cells are also capable to target both LCN2 forms into exosomes (Figure 8).

### Functional relevance of *N*-glycosylation

We and others have previously shown that LCN2 is a kind of “help me” signal that is upregulated during tissue inflammation and in response to cellular stress (Asimakopoulou et al., 2016a; Wieser et al., 2016). We have suggested LCN2 to be critically involved in promoting neutrophil recruitment into the inflamed tissue (Asimakopoulou et al., 2016b). Because *Lcn2* deficient mice showed reduced neutrophil infiltration, liver injury and hepatic steatosis during alcoholic liver disease, it was speculated that the pharmacological neutralization of LCN2 might be of promise in the respective disease (Asimakopoulou et al., 2016b). In addition, there are many other experimental and clinical findings showing that neutralization of hepatic LCN2 might evolve beneficial effects on liver homeostasis (Asimakopoulou et al., 2016b).

We here demonstrated that epithelial cells, including primary hepatocytes, human lung carcinoma cell line A549, and prostate cancer cell line PC-3 can target LCN2 to exosome vesicles, irrespectively if it is glycosylated or not. We actually do not know about the functional significance of LCN2 glycosylation, but the high conservation of the N-glycosylation site during evolution suggests proper glycosylation of the respective amino acid (i.e., Asn^85^) in LCN2 should have important biological or biochemical implications. Rudd and co-workers have shown by use of MALDI-TOF-MS analysis that 80% of the sugars were mono-sialylated, and many contained an outer arm fucose residue and further that human LCN2 contains a population of glycans carrying a single fucose on one antenna, but none on the core (Rudd et al., 1999). A more recent study performed by Zhao and coworkers analyzing the C87S mutant recombinant LCN2 protein purified from CHO cells confirmed the complex *N*-glycosylation pattern (Zhao et al., 2010). In the mentioned study, the mutant recombinant LCN2 contained six deconvoluted mass spectra in which the pattern of *N*-glycosylation was very complex and heterogeneous. They comprised triantennary, tetraantennary glycans, hybrid type, and a few high mannose type glycans. Interestingly, some of the LCN2 samples identified carried one additional sialic acid or two additional fucoses. The authors speculated that the different glycan patterns observed in the different LCN2 entities may be due to the different harvest times in cell culture (Zhao et al., 2010). Altogether, these studies show that *N*-glycosylation at Asn^85^ is rather complex. *In vivo* studies investigating LPS-induced inflammation or unilateral ureteral obstruction resulted in the identification of several isoforms of urinary LCN2 showing molecular weight variation due to differences in *N*-glycan structure (Fujiwara et al., 2016). Strikingly, the glycosylation pattern of the LCN2 species varied between the analyzed models suggesting that the occurrence of specific glycosylated urinary LCN2 isoforms depends on the pathological condition (Fujiwara et al., 2016).

On the long term, the existence of multiple glycosylated forms of LCN2 may widen the spectrum of diagnostics. Changes in the *N*-linked glycome have been reported in many disease conditions, including cancer and chronic inflammation (Arnold et al., 2008). Therefore, it is reasonable that the occurrence of different *N*-glycosylated forms of LCN2 reflect systemic or organ specific alterations and worth to be tested as novel potential biomarkers. In regard to LCN2, the organ or tissue of synthesis might predict its pattern of glycosylation. LCN2 behaves like an acute phase response protein, which is induced in mice after injection of turpentine and dexamethasone (Liu and Nilsen-Hamilton, 1995). Many other acute phase proteins are glycoproteins. In addition to changes in the serum levels of these proteins, the glycan structures attached to these molecules can be modified in response to this complex systemic early-defense reaction (Muller, 2014). In line, first reports have shown that determination of LCN2 in urinary exosomes is a better predictor of kidney dysfunction after kidney transplantation than other urinary fractions (Alvarez et al., 2013).

In the liver, LCN2 is drastically induced during inflammation (Borkham-Kamphorst et al., 2011). As a consequence, inflammatory cells enter the inflamed tissue (Asimakopoulou et al., 2016a; Wieser et al., 2016). During leukocyte extravasation (diapedesis), a well-orchestrated program occurs in which chemoattraction, rolling adhesion, tight adhesion and endothelial transmigration occurs (Muller, 2014). As a major constituent of neutrophils, the biological activity of LCN2 or its capacity to bind to metalloproteinases (e.g. MMP-9) might be modified by *N*-glycosylation. Interestingly, the *N*-glycosylation site is in close proximity to a conserved cysteine residue (Supplementary Figure 7) forming a disulfide bond with an as yet unidentified cysteine residue in MMP-9 (Strong et al., 1998; Cabedo Martinez et al., 2016). The formation of this heteromer protects MMP-9 from degradation. Masking or steric hindrance by *N*-glycosylation might prevent binding of these two neutrophil constituents. However, this is not really likely. A landmark paper from the year 2001 comparing LCN2 isolated from granulocytes with recombinant LCN2 forms expressed by the yeast *Pichia pastoris* or by *Escherichia coli* had already shown that the carbohydrate moiety is not essential for the biological activity of LCN2 in accelerating the direct activation of the promatrix metalloproteinases proMMP-9 and pro-MMP-8 (Tschesche et al., 2001).

### Alternative functions of *N*-glycosylation

Alternatively, the glycosylation prevents direct forming of LCN2 homodimers or the formation of internal disulfide bonds. Hydrophobic cluster analysis (HCA) suggests that the respective Asn carrying the *N*-glycosylation is embedded in a highly hydrophobic surrounding (Supplementary Figure 8). Addition of net charges to this surrounding might prevent neutrophil chemotaxis by increasing LCN2's affinity for cytokines or chemokines, thereby inhibiting signaling through their specific receptor pathways. Such activities are already known for other acute phase proteins. While for example glycosylated α1-Antitrypsin modulates neutrophil chemotaxis by binding to IL-8 and preventing signaling through the CXCR1 receptor pathway, the non-glycosylated form did not possess the same anti-inflammatory activities (Bergin et al., 2010).

Another possibility is that the attached sialic acids in the siderophore LCN2 could act as direct chemoattractants for bacteria. Many bacteria not only use sialic acid as a nutrient, but also incorporate sialic acid in their cell surface, helping them to evade or resist from components of the innate immune response of the host (Severi et al., 2007). Such a biological sugar trap in combination with the affinity of LCN2 to bacterial siderophores might be important in the innate immune response to bacterial infections.

A recent study showed bacterially expressed LCN2 has the capacity to form complexes with the siderophore Enterochelin (also known as Enterobactin) proving that the *N*-glycosylation is not necessary for capturing catecholates (Barasch et al., 2016). Concurrently, Enterochelin is a potent inhibitor of myeloperoxidase (MPO) most abundantly expressed in neutrophil granulocytes, which produce hypohalous acids comprising antimicrobial activity (Singh et al., 2015). LCN2 can rescue and maintain MPO function by physically interacting with Enterochelin (Singh et al., 2015). Therefore, it is also possible that the *N*-glycosylated form of LCN2 is more suitable in preserving MPO function by binding tighter to Enterochelin.

We must admit that there are still several other possibilities how *N*-glycosylation of LCN2 might fine tune processes counteracting bacterial uptake of iron-loaded siderophores. Some of them had been recently highlighted in a review suggesting the siderophore-binding protein LCN2 acts in a “Tug-of-war” against bacterial siderophores (Wilson et al., 2016). In this scenario the attached sugar tree may be one of the required structural elements in defining the molecular interface necessary to prevent pathogens from acquiring iron through their high-affinity siderophores.

Based on all these considerations, it will be in future of major interest to compare the biological activities of glycosylated and unglycosylated LCN2 *in vivo*. The finding that LCN2 glycosylation is effectively blocked by administration of tunicamycin *in vitro* will offer the opportunity to prepare large batches of purified unglycosylated LCN2 for *in vitro* and *in vivo* experimentation. This is important, because the direct application of tunicamycin in liver disease models is not possible. The lethal doses of tunicamycin in mice were determined to LD_50_ of 2.0 mg/kg and LD_100_ of 3.5 mg/kg body weight with major pathological manifestations of tunicamycin toxicity occurring first and foremost in the liver (Morin and Bernacki, 1983). Therefore, the direct *in vivo* application of this drug for blockade of LCN2 glycosylation is too harmful. The finding that tunicamycin blocks LCN2 glycosylation will offer a suitable alternate strategy to prepare both LCN2 variant forms for a wealth of applications.

## Conclusion

LCN2 is a secreted protein produced in high quantities in hepatocytes during inflammation. It is post-translationally modified at an evolutionarily conserved *N*-glycosylation site, possibly relevant to determine biochemical characteristics of LCN2. The glycosylation of LCN2 can be effectively blocked by application of tunicamycin without preventing its proper secretion or exosome targeting. Our study provides a valuable starting point for future work aiming to comparatively analyze the biological characteristics of the glycosylated and unglycosylated LCN2 variants.

## Author contributions

All authors have materially participated in the research of this study and approved the final version of this article. EB-K, EVdL, EB, SM, and RW performed experiments. RW designed the study and wrote the manuscript.

### Conflict of interest statement

The authors declare that the research was conducted in the absence of any commercial or financial relationships that could be construed as a potential conflict of interest.

## Footnotes

^1^The human protein atlas. www.proteinatlas.org/humanproteome/secretome (Accessed March 19, 2018).

^2^Clustal Omega. http://www.ebi.ac.uk/Tools/msa/clustalo/ (Accessed March 5, 2018).

^3^SignalP 4.1 Server. Department of Bio and Health Informatics. http://www.cbs.dtu.dk/services/SignalP/ (Accessed March 19, 2018).

^4^NetNGlyc 1.0 Server. Department of Bio and Health Informatics. http://www.cbs.dtu.dk/services/NetNGlyc/ (Accessed March 19, 2018).

^5^Hydrophobic cluster analysis. http://mobyle.rpbs.univ-paris-diderot.fr/cgi-bin/portal.py?form=HCA#forms::HCA (Accessed March 19, 2018).

^6^Santa Cruz Biotechnology, Inc., Datasheet for Alix (1A12): sc-53540. https://datasheets.scbt.com/sc-53540.pdf (Accessed March 19, 2018).

## Supplementary material

Supplementary Figure 1 Multiple sequence alignment of LCN2 from various species and prediction of *N*-glycosylation. **(A)** LCN2 protein sequences from human (HS), rhesus macaque (MA), Chinese hamster (CG), mouse (MM), rat (RN), cheetah (AJ), cattle (BT) horse (EC), and Malayan Pangolin (MJ) were aligned using Clustal Omega. An asterisk (^*^) indicates a position with fully conserved residue, a colon (:) indicates a conservation of amino acids with strongly similar properties and a period (•) indicates a conservation of amino acids with weakly similar properties. Amino acid positions are given in the right margin. Potential *N*-glycosylation sites determined by the NetNGlyc *N*-glycosylation site predictor are depicted in reversed type, the cysteine residues (Cys 96 and Cys 195 in human LCN2) proposed to be involved in the formation of LCN2 homodimers, oligomers, or intramolecular disulfide bounds are marked in red letters, and secretory signal peptides are boxed in yellow. **(B)** The primary LCN2 protein sequences of human (*Homo sapiens*), mouse (*Mus musculus*) and cheetah (*Acinonyx jubatus*) were analyzed for potential *N*-glycosylation sites using the NetNGlyc *N*-Glycosylation site predictor. The individual graphs illustrate predicted *N*-glyc sites across the protein chain in which the x-axis represents amino acid positions from N- to C-terminus. A position with a potential glycosylation site (*vertical line*) crossing the threshold (*horizontal line* at 0.5) is predicted as glycosylated.

Supplementary Figure 2 Stimulation of LCN2 expression in TW60 cells. Cell extracts and conditioned media prepared from TW60 cells left untreated (control) or stimulated with 400 ng/mL LPS or 2.5 ng/mL IL-1β in the presence or absence of tunicamycin (TUN) were analyzed for expression of LCN2. GAPDH expression served as control to demonstrate equal gel loading in cell extracts.

Supplementary Figure 3 Lack of LCN2 expression in NB4 cells. **(A)** Cell extracts and **(B)** conditioned media of NB4 cells stimulated with LPS, tunicamycin (TUN), LPS and TUN or left untreated were analyzed for expression of LCN2. In this experiment, cells were cultured in medium containing 10% FCS or serum-free medium. Ponceau S stain served as control to demonstrate integrity of protein samples. Please note, even after long exposure of membranes, LCN2 was not detectable in these cells.

Supplementary Figure 4 Lack of LCN2 expression in cell extracts of dHL-60 and dNB4 cells. Cell extracts of differentiated dHL-60 and dNB4 cells stimulated with LPS (200 ng/mL) or LPS and different concentrations of tunicamycin (TUN, 50 or 100 μg/mL) were analyzed for expression of LCN2 and MPO. A cell extract isolated from IL-1β-stimulated HepG2 cells served as control. Equal protein loading was demonstrated by Ponceau S stain and probing with an antibody specific for β-actin. Please note, although cell extracts were positive for MPO, LCN2 was not detectable.

Supplementary Figure 5 Lack of LCN2 expression in conditioned media of dHL-60 and dNB4 cells. Conditioned culture media of differentiated dHL-60 and dNB4 cells stimulated with LPS or LPS and different concentrations of tunicamycin (TUN) were analyzed for expression of LCN2. A cell extract isolated from IL-1β-stimulated HepG2 cells served as control. Equal protein loading was demonstrated by Ponceau S stain. Please note, even after long exposure of the membrane, LCN2 was not detectable.

Supplementary Figure 6 Expression of LCN2 in A549 and PC-3 cells. **(A)** Cell extracts and **(B)** conditioned media of A549 and PC-3 cells left untreated (control) or stimulated with IL-1β, tunicamycin (TUN), IL-1β and TUN were analyzed for expression of LCN2. GAPDH expression served as control to demonstrate equal gel loading in cell extracts.

Supplementary Figure 7 LCN2 protein structure. LCN2 belongs to the lipocalin family sharing a typical eight-stranded, anti-parallel, symmetrical β-barrel fold structure. The depicted structure was generated using the Ribbons XP software (version 3.0) and X-ray diffraction coordinates of an engineered human apo-form of LCN2 resolved at resolution 2.0 Å which are deposited under accession number 3BX8 in the RCSB Protein Data Bank^7^. A size marker (10 Å) is given and the position of Asn^85^ and Cys^96^ are indicated. The numbering of amino acids refers to the start Met^1^ of human LCN2 (cf. Supplementary Figure 1).

Supplementary Figure 8 Hydrophobic cluster analysis. The protein sequences of human, mouse, rat, bovine, Chinese hamster, horse, pangolin, rhesus macaque, and cheetah were subjected to a hydrophobic cluster analysis. Symbols are used to represent amino acids with peculiar structural properties (red star for proline, black diamond for glycine, square and dotted square for threonine and serine, respectively). The positions of the glycosylated asparagines in the LCN2 of each species are indicated by an arrow. Please note, that this residue is embedded in a highly hydrophobic surrounding in all species.

Supplementary Video 1 NTA measurement for size determination of exosomes isolated from conditioned media of hepatocytes cultured in the presence of DMSO as vehicle for 24 h. Isolation of exosomes was done as described in the Materials and Methods section (see Method for Isolation of Exosomes from Conditioned Media).

Supplementary Video 2 NTA measurement for size determination of exosomes isolated from conditioned media of hepatocytes cultured in the presence of 0.5 μg/mL tunicamycin for 24 h. Isolation of exosomes was done as described in the Materials and Methods section (see Method for Isolation of Exosomes from Conditioned Media).
