# Supplementary figures and images for: N-Glycosylation of Lipocalin 2 Is Not Required for Secretion or Exosome Targeting
Source: Front Pharmacol. 2018 Apr 25;9:426. doi: 10.3389/fphar.2018.00426 (PMC5932398; doi:10.3389/fphar.2018.00426)

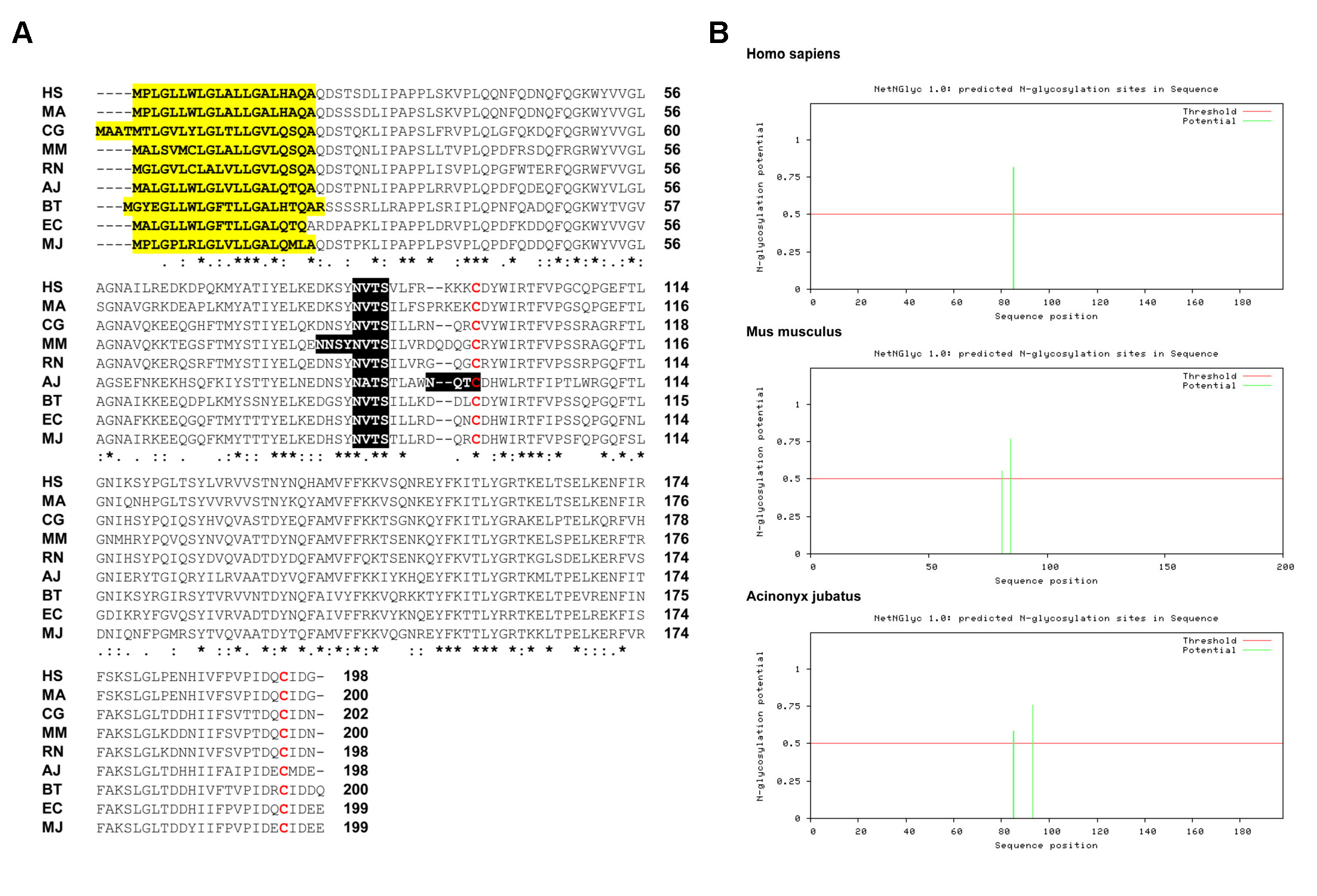

Supplement: Supplementary Figure 1 — Multiple sequence alignment of LCN2 from various species and prediction of N-glycosylation. (A) LCN2 protein sequences from human (HS), rhesus macaque (MA), Chinese hamster (CG), mouse (MM), rat (RN), cheetah (AJ), cattle (BT) horse (EC), and Malayan Pangolin (MJ) were aligned using Clustal Omega. An asterisk (*) indicates a position with fully conserved residue, a colon (:) indicates a conservation of amino acids with strongly similar properties and a period (•) indicates a conservation of amino acids with weakly similar properties. Amino acid positions are given in the right margin. Potential N-glycosylation sites determined by the NetNGlyc N-glycosylation site predictor are depicted in reversed type, the cysteine residues (Cys 96 and Cys 195 in human LCN2) proposed to be involved in the formation of LCN2 homodimers, oligomers, or intramolecular disulfide bounds are marked in red letters, and secretory signal peptides are boxed in yellow. (B) The primary LCN2 protein sequences of human (Homo sapiens), mouse (Mus musculus) and cheetah (Acinonyx jubatus) were analyzed for potential N-glycosylation sites using the NetNGlyc N-Glycosylation site predictor. The individual graphs illustrate predicted N-glyc sites across the protein chain in which the x-axis represents amino acid positions from N- to C-terminus. A position with a potential glycosylation site (vertical line) crossing the threshold (horizontal line at 0.5) is predicted as glycosylated. [file Image_1.JPEG]

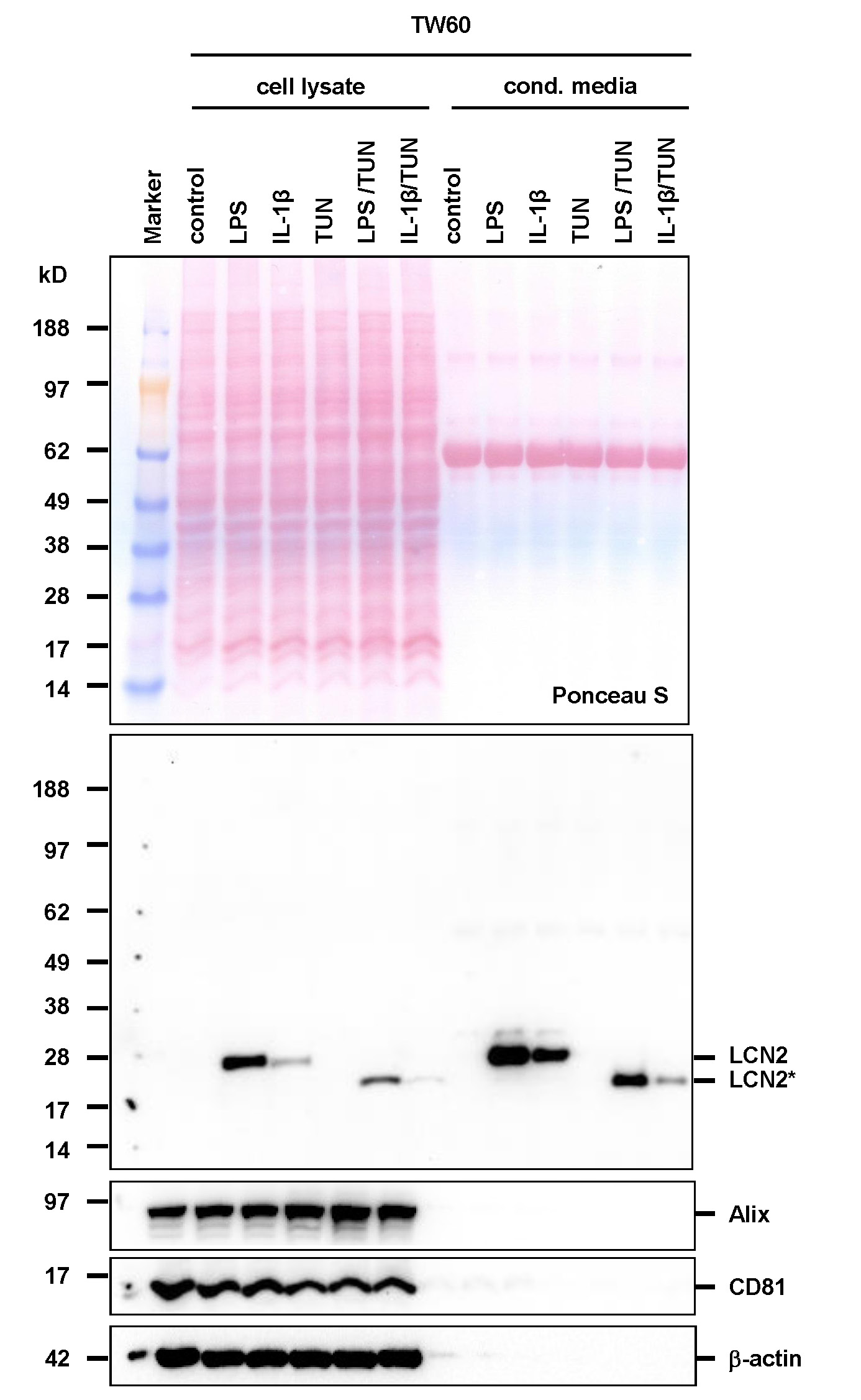

Supplement: Supplementary Figure 2 — Stimulation of LCN2 expression in TW60 cells. Cell extracts and conditioned media prepared from TW60 cells left untreated (control) or stimulated with 400 ng/mL LPS or 2.5 ng/mL IL-1β in the presence or absence of tunicamycin (TUN) were analyzed for expression of LCN2. GAPDH expression served as control to demonstrate equal gel loading in cell extracts. [file Image_2.JPEG]

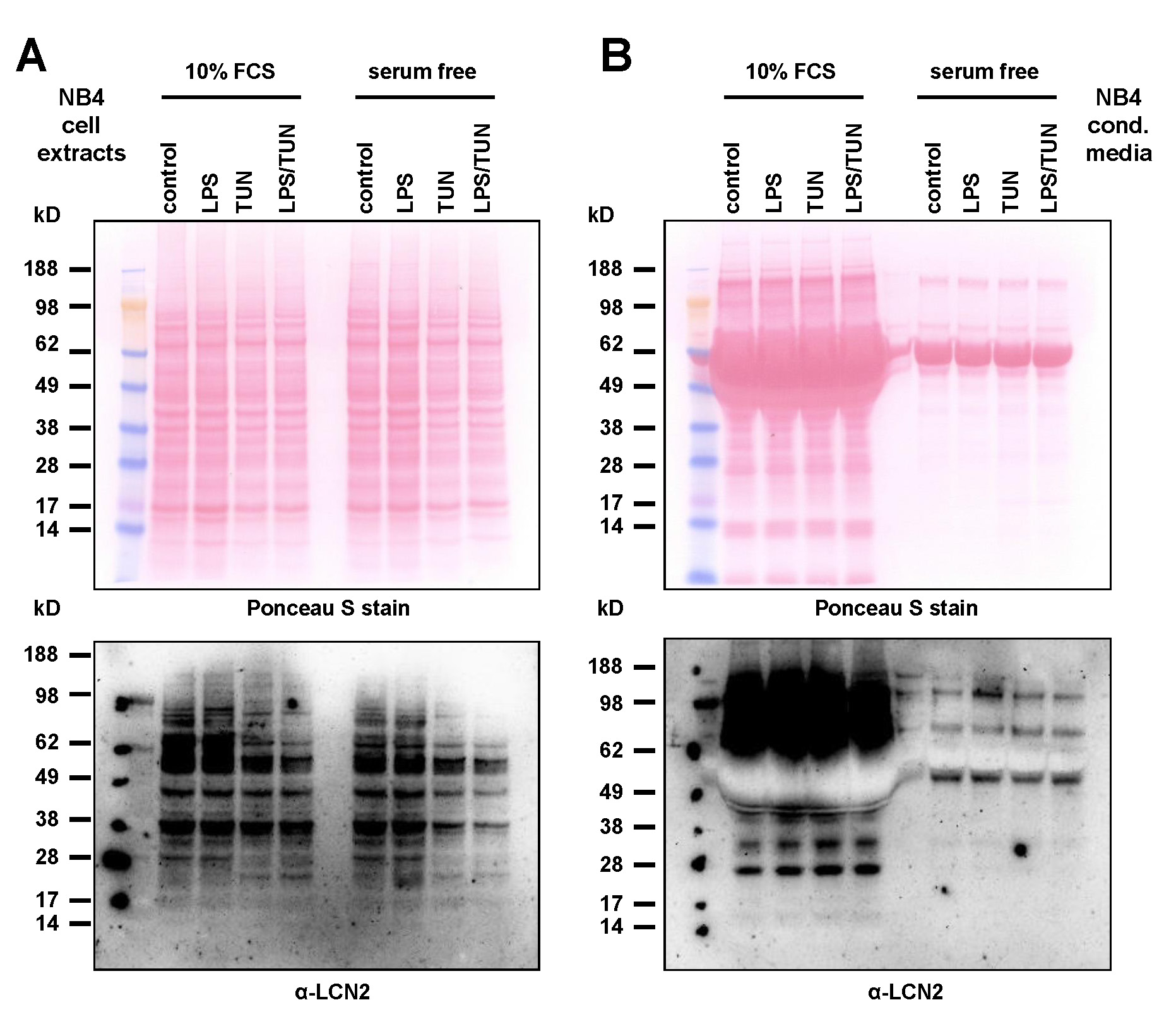

Supplement: Supplementary Figure 3 — Lack of LCN2 expression in NB4 cells. (A) Cell extracts and (B) conditioned media of NB4 cells stimulated with LPS, tunicamycin (TUN), LPS and TUN or left untreated were analyzed for expression of LCN2. In this experiment, cells were cultured in medium containing 10% FCS or serum-free medium. Ponceau S stain served as control to demonstrate integrity of protein samples. Please note, even after long exposure of membranes, LCN2 was not detectable in these cells. [file Image_3.JPEG]

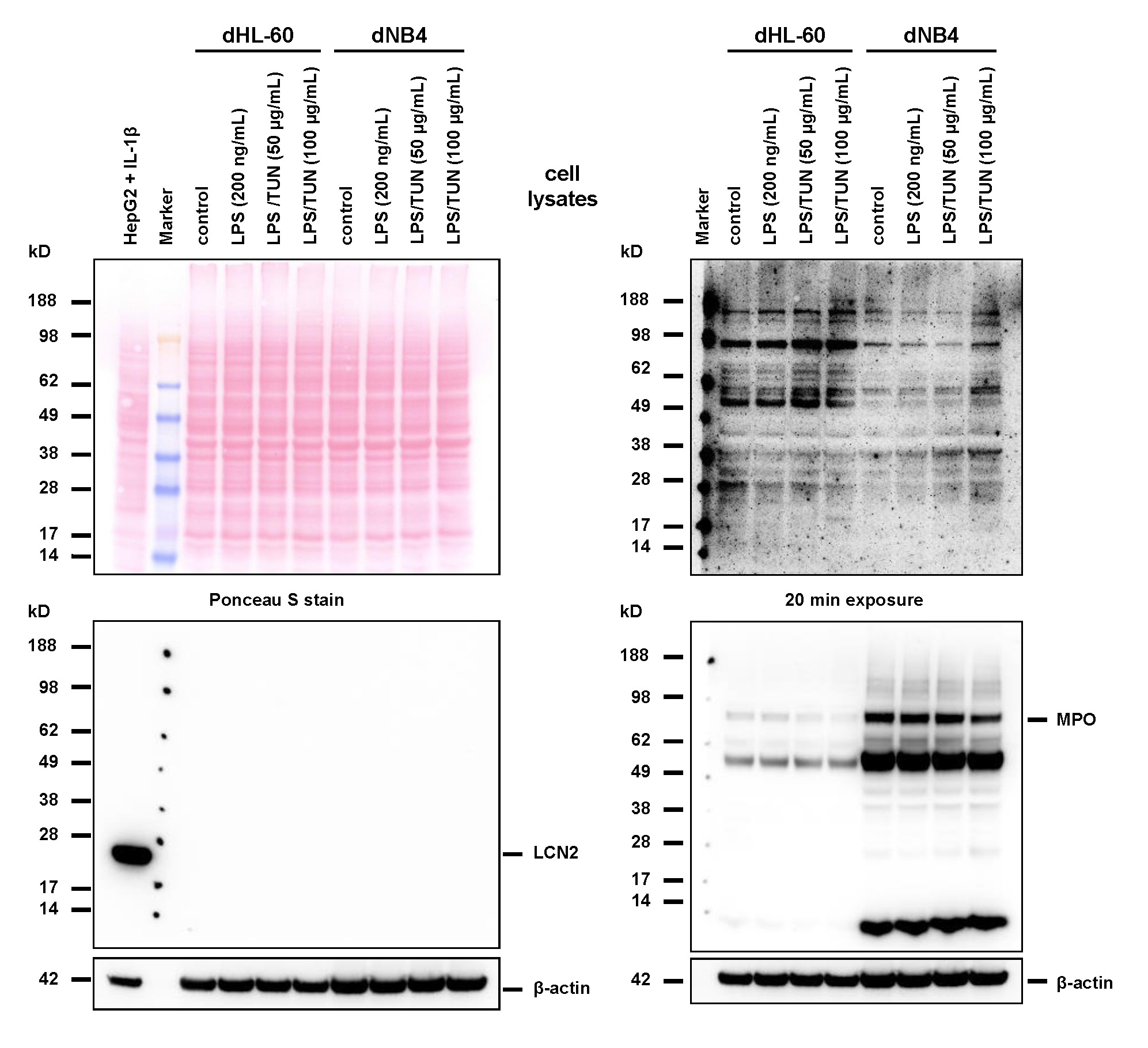

Supplement: Supplementary Figure 4 — Lack of LCN2 expression in cell extracts of dHL-60 and dNB4 cells. Cell extracts of differentiated dHL-60 and dNB4 cells stimulated with LPS (200 ng/mL) or LPS and different concentrations of tunicamycin (TUN, 50 or 100 μg/mL) were analyzed for expression of LCN2 and MPO. A cell extract isolated from IL-1β-stimulated HepG2 cells served as control. Equal protein loading was demonstrated by Ponceau S stain and probing with an antibody specific for β-actin. Please note, although cell extracts were positive for MPO, LCN2 was not detectable. [file Image_4.JPEG]

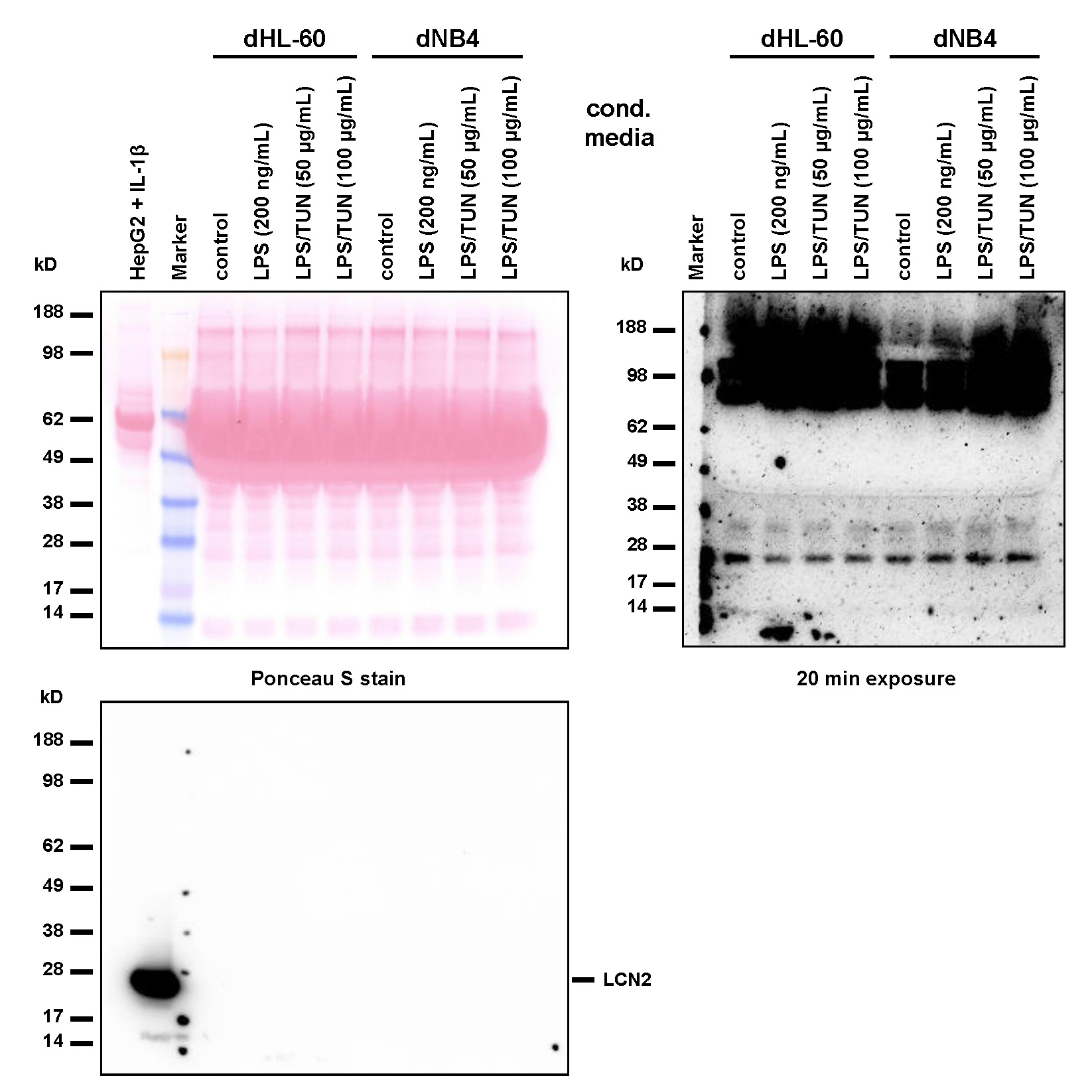

Supplement: Supplementary Figure 5 — Lack of LCN2 expression in conditioned media of dHL-60 and dNB4 cells. Conditioned culture media of differentiated dHL-60 and dNB4 cells stimulated with LPS or LPS and different concentrations of tunicamycin (TUN) were analyzed for expression of LCN2. A cell extract isolated from IL-1β-stimulated HepG2 cells served as control. Equal protein loading was demonstrated by Ponceau S stain. Please note, even after long exposure of the membrane, LCN2 was not detectable. [file Image_5.JPEG]

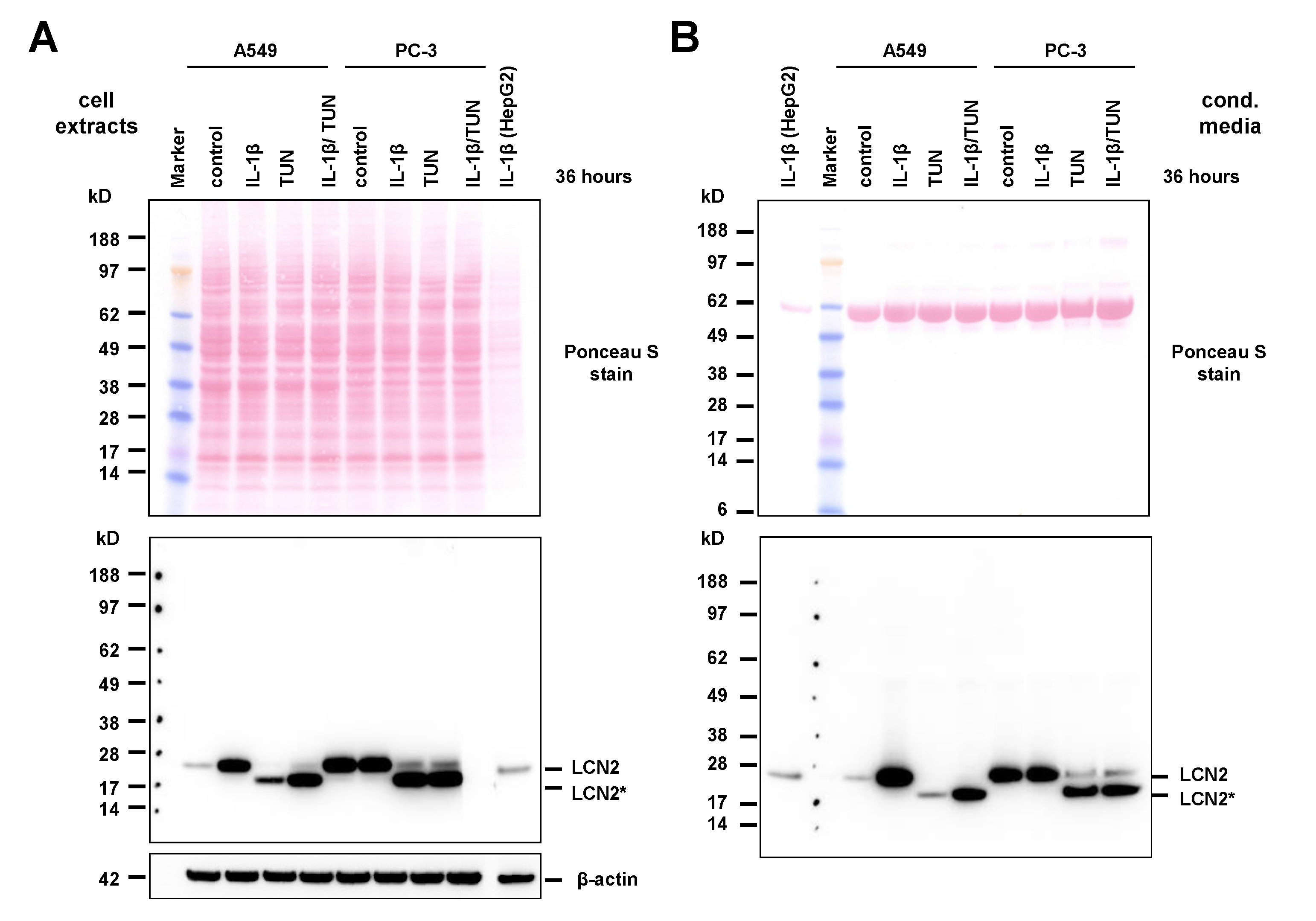

Supplement: Supplementary Figure 6 — Expression of LCN2 in A549 and PC-3 cells. (A) Cell extracts and (B) conditioned media of A549 and PC-3 cells left untreated (control) or stimulated with IL-1β, tunicamycin (TUN), IL-1β and TUN were analyzed for expression of LCN2. GAPDH expression served as control to demonstrate equal gel loading in cell extracts. [file Image_6.JPEG]

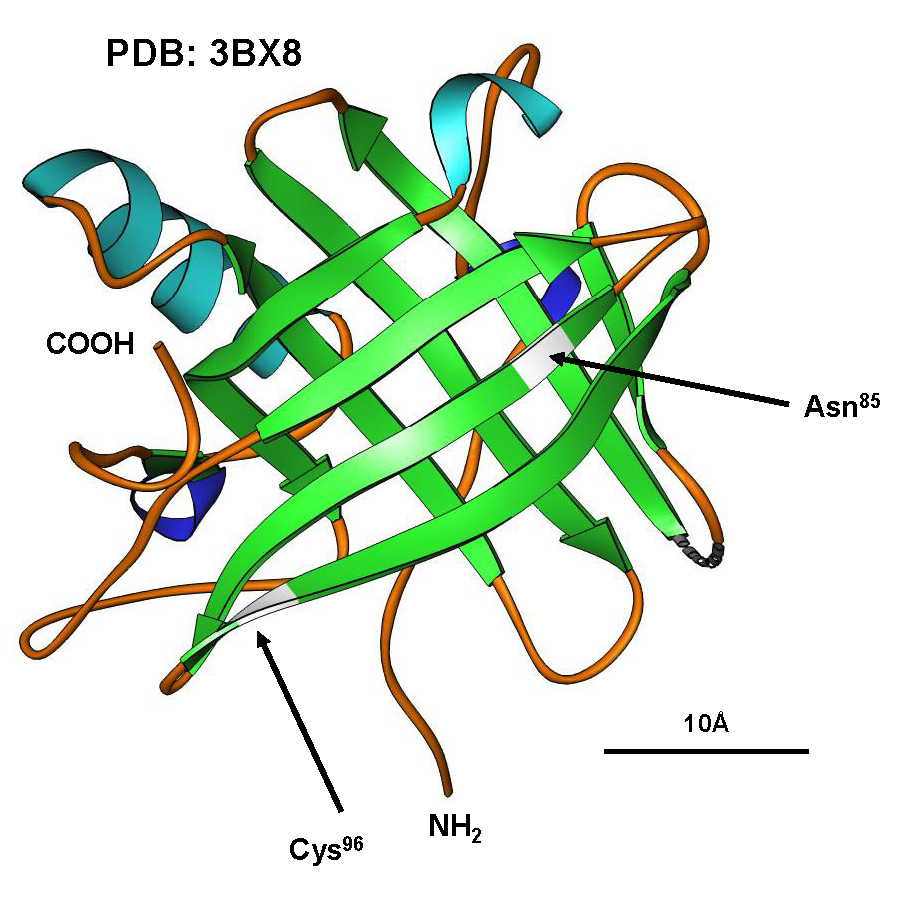

Supplement: Supplementary Figure 7 — LCN2 protein structure. LCN2 belongs to the lipocalin family sharing a typical eight-stranded, anti-parallel, symmetrical β-barrel fold structure. The depicted structure was generated using the Ribbons XP software (version 3.0) and X-ray diffraction coordinates of an engineered human apo-form of LCN2 resolved at resolution 2.0 Å which are deposited under accession number 3BX8 in the RCSB Protein Data Bank7. A size marker (10 Å) is given and the position of Asn85 and Cys96 are indicated. The numbering of amino acids refers to the start Met1 of human LCN2 (cf. Supplementary Figure 1). [file Image_7.JPEG]

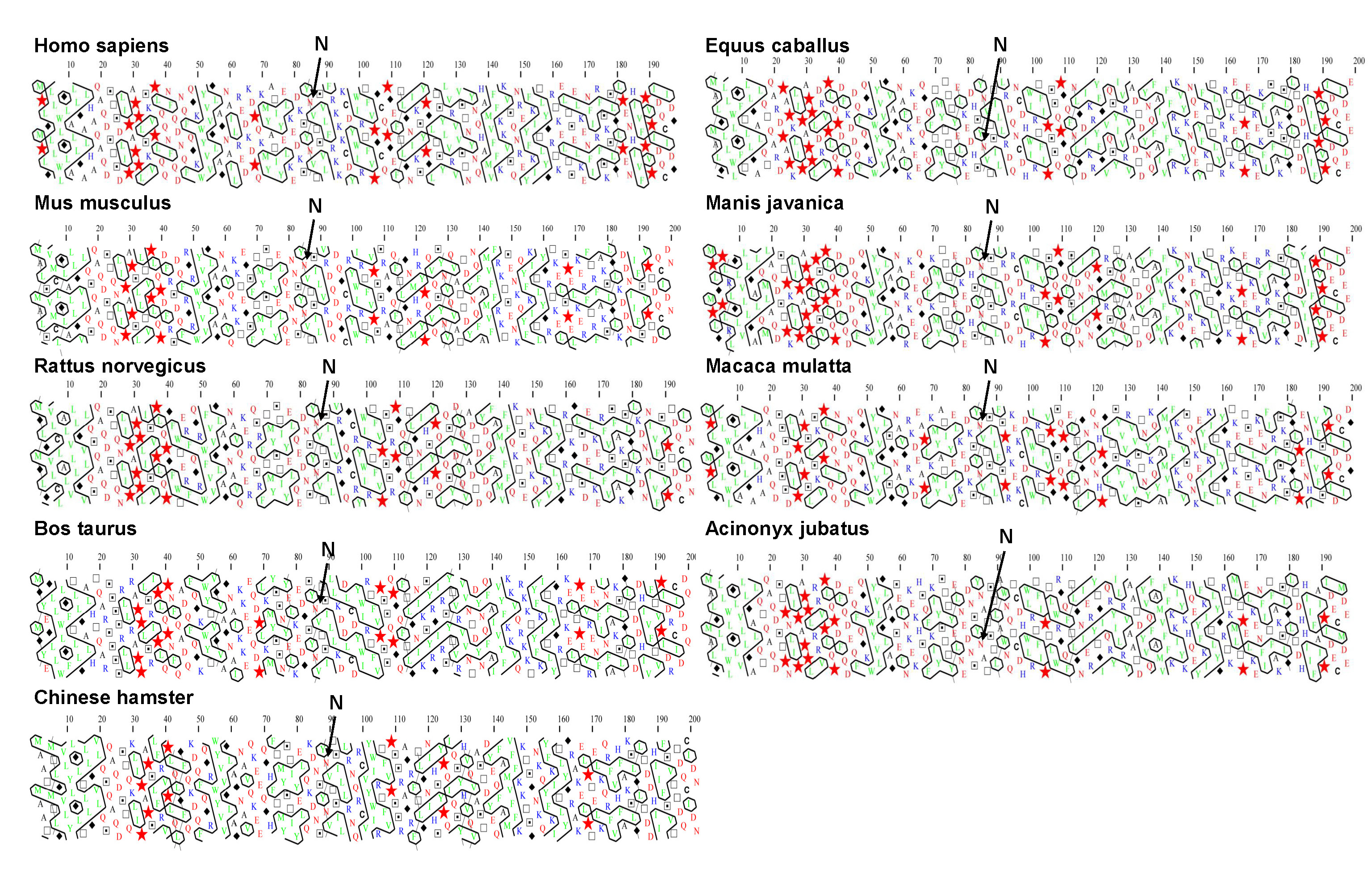

Supplement: Supplementary Figure 8 — Hydrophobic cluster analysis. The protein sequences of human, mouse, rat, bovine, Chinese hamster, horse, pangolin, rhesus macaque, and cheetah were subjected to a hydrophobic cluster analysis. Symbols are used to represent amino acids with peculiar structural properties (red star for proline, black diamond for glycine, square and dotted square for threonine and serine, respectively). The positions of the glycosylated asparagines in the LCN2 of each species are indicated by an arrow. Please note, that this residue is embedded in a highly hydrophobic surrounding in all species. [file Image_8.JPEG]
